# The role of endometrial scratching prior to in vitro fertilization: an updated systematic review and meta-analysis

**DOI:** 10.1186/s12958-023-01141-2

**Published:** 2023-10-02

**Authors:** Maria Chrysoula Iakovidou, Efstratios Kolibianakis, Leonidas Zepiridis, Christos Venetis

**Affiliations:** 1 Unit for Human Reproduction, Medical School, 1st Department of Obstetrics & Gynecology, Papageorgiou General Hospital, Peripheral Road, Nea Efkarpia, 56430 Thessaloniki, Greece; 2 Centre for Big Data Research in Health, Faculty of Medicine & Health, University of New South Wales, Sydney, Australia

**Keywords:** Endometrial scratching, Endometrial injury, In vitro fertilization, Reproductive outcomes, Live birth

## Abstract

**Research question:**

To evaluate the role of endometrial scratching performed prior to an embryo transfer cycle on the probability of pregnancy compared to placebo/sham or no intervention.

**Design:**

A computerized literature (using a specific search strategy) search was performed across the databases MEDLINE, EMBASE, COCHRANE CENTRAL, SCOPUS and WEB OF SCIENCE up to June 2023 in order to identify randomized controlled trials (RCTs) evaluating the effect of endometrial scratching prior to an embryo transfer cycle on the probability of pregnancy, expressed either as live birth, ongoing pregnancy or clinical pregnancy (in order of significance) compared to placebo/sham or no intervention. Data were pooled using random-effects or fixed-effects model, depending on the presence or not of heterogeneity. Heterogeneity was assessed using the *I*^2^ statistic. Subgroup analyses were performed based on the population studied in each RCT, as well as on the timing and method of endometrial biopsy. Certainty of evidence was assessed using the GRADEPro tool.

**Results:**

The probability of live birth was significantly higher in embryo transfer cycles after endometrial scratching as compared to placebo/sham or no intervention (relative risk-RR: 1.12, 95% CI: 1.05–1.20; heterogeneity: I^2^=46.30%, *p*<0.001, 28 studies; low certainty). The probability of ongoing pregnancy was not significantly difference between the two groups (RR: 1.07, 95% CI: 0.98–1.18; heterogeneity: I^2^=27.44%, p=0.15, 11 studies; low certainty). The probability of clinical pregnancy was significantly higher in embryo transfer cycles after endometrial scratching as compared to placebo/sham or no intervention (RR: 1.12, 95% CI: 1.06–1.18; heterogeneity: I^2^=47.48%, *p*<0.001, 37 studies; low certainty).

A subgroup analysis was performed based on the time that endometrial scratching was carried out. When endometrial scratching was performed during the menstrual cycle prior to the embryo transfer cycle a significantly higher probability of live birth was present (RR: 1.18, 95% CI:1.09-1.27; heterogeneity: I^2^=39.72%, *p*<0.001, 21 studies; moderate certainty). On the contrary, no effect on the probability of live birth was present when endometrial injury was performed during the embryo transfer cycle (RR: 0.87, 95% CI: 0.67-1.15; heterogeneity: I^2^=65.18%, *p*=0.33, 5 studies; low certainty).

In addition, a higher probability of live birth was only present in women with previous IVF failures (RR: 1.35, 95% CI: 1.20-1.53; heterogeneity: I^2^=0%, *p*<0.001, 13 studies; moderate certainty) with evidence suggesting that the more IVF failures the more likely endometrial scratching to be beneficial (*p*=0.004). The number of times endometrial scratching was performed, as well as the type of instrument used did not appear to affect the probability of live birth.

**Conclusions:**

Endometrial scratching during the menstrual cycle prior to an embryo transfer cycle can lead to a higher probability of live birth in patients with previous IVF failures.

**PROSPERO registration:**

PROSPERO CRD42023433538 (18 Jun 2023)

**Supplementary Information:**

The online version contains supplementary material available at 10.1186/s12958-023-01141-2.

## Introduction

Success rate following assisted reproductive technologies (ART) remain low. This has stimulated researchers worldwide to investigate the two main factors responsible for the achievement of pregnancy, namely embryo quality and endometrial receptivity. Regarding the latter, a variety of strategies have been proposed to enhance endometrial receptivity and thus increase the probability of pregnancy after ART.

Endometrial scratching is a procedure undertaken to purposely disrupt the endometrium in women aiming to get pregnant, since this intervention has been suggested to increase the chance of embryo implantation [1]. A considerable number of relevant observational and randomized-controlled trials (RCTs) have been published. These have been summarized in systematic reviews and meta-analyses, which suggested the presence of a positive effect of endometrial scratching on the probability of pregnancy [2, 3].

Due to these initial findings, endometrial scratching was implemented as a standard procedure prior to IVF in many fertility clinics throughout the world [4]. However, a large RCT published in 2019 suggested no benefit from the procedure [5], and this led the scientific community to revisit the idea of endometrial scratching [6]. The most recent Cochrane systematic review and meta-analysis published in 2021 included 38 trials and suggested that the effect of endometrial injury on the probability of live birth and clinical pregnancy among women undergoing IVF is unclear [7]. In the same year, a large multi-centered randomised controlled trial (SCRaTCH) suggested, marginally non-statistically significant, but clinically important differences of endometrial scratching on live birth rates [8, 9]. This once again fuelled the controversy regarding the potential benefit of endometrial scratching, pointing to the need for further evaluation [10]. In the presence of additional RCTs published after 2021, this systematic review and meta-analysis will attempt to clarify the contentious role of endometrial scratching prior to in vitro fertilization on the probability of pregnancy, expressed as live birth, ongoing or clinical pregnancy in specific subgroups, depending on the population studied and the method of endometrial scratching used.

## Materials and methods

### Search strategy

A computerized literature search in MEDLINE, EMBASE, Cochrane CENTRAL, Scopus and Web of Science covering the period until June 2023 was performed independently by two reviewers (MCI and CAV) aiming to identify RCTs that evaluated the following research question: does endometrial scratching undertaken prior to an IVF cycle increase the probability of live birth compared to or placebo/sham or no intervention? For this purpose, the free-text search terms [(endometr*) AND (scratch* OR injur* OR traum* OR biops* OR sampl* OR damag* OR activat* OR stimulat*)] AND [(in vitro fertilization) OR (in vitro fertilisation) OR IVF OR ICSI OR (intracytoplasmic sperm injection) OR (assisted reproduct*) OR (assisted conception)] AND [(random* OR (clinical trial) OR placebo OR sham)] were used. Additionally, the citation lists of relevant publications and previous systematic reviews were hand-searched. In case of overlapping reports (i.e. reports of the same RCT), the more extensive one was included.

No language limitations were applied. Authors of this article report no conflict of interest with any commercial entity, whose products are described, reviewed, evaluated, or compared in this study.

### Selection of studies

Criteria for inclusion/exclusion of studies were established prior to the literature search and the protocol was published to the PROSPERO registry (CRD42023433538). Studies had to fulfill the following criteria for eligibility: a) randomized controlled trials comparing patients who underwent endometrial scratching prior to embryo transfer compared with those who did not, regardless of the type of procedure used to scratch the endometrium and the protocols of ovarian stimulation for IVF and/or endometrial preparation. Selection of the studies was performed independently by two of the reviewers (MCI and CAV). Any disagreement was resolved by discussion.

### Data extraction

The following data were extracted from each of the eligible studies: demographic (type of study, citation data, country, study period, number of patients included, methodological (randomization method, allocation concealment, blinding, whether power analysis was performed, primary outcome assessed, whether there was financial support for the trial, whether there was a protocol registration) (Table 1), procedural (inclusion criteria, exclusion criteria, type of embryo transfer (fresh/ frozen), method of endometrial injury, timing of intervention, instrument used, control/ type of intervention, timing of control intervention, other interventions, definitions of pregnancy outcomes) (Table 2), outcome data (live birth rate per randomized patient, ongoing pregnancy per randomized patient, clinical pregnancy rate per randomized patient, cumulative live birth rate, miscarriage rate, ectopic pregnancy rate, multiple pregnancy rate, pain during the procedure using Visual Analogue Scale (VAS) measures, adverse events [e.g., infection, uterine perforation, uterine adhesions, bleeding]). Any disagreement was resolved unanimously by discussion. An effort was made to contact the authors of the eligible studies to retrieve missing or additional information, where necessary.

**Table 1:** Methodological characteristics of eligible studies

**Study, country of origin, number of centers, journal or meeting**	**Study period**	**Number of patients randomized**	**Randomization method**	**Allocation concealment**	**Blinding**	**Prospective power analysis performed**	**Primary outcome assessed**	**Financial Support**	**Protocol Registration**	**Authors contacted**
Karim Zadeh et al., (2008) [11], Iran, single center, Human Reproduction	Not reported	160 (endometrial scratching: 80, control: 80)	Not reported	Not reported	Not reported	Not reported	Implantation (definition not reported)	Not reported	Not reported	Attempted; No response
Karimzadeh et al., (2009) [12], Iran, single center, Australian and New Zealand Journal of Obstetrics and Gynaecology	Not reported	115 (endometrial scratching: 58, control: 57)	Manual randomization (drawing a piece of paper from a bag)	Not reported	Not reported	Yes	Pregnancy (definition not reported)	Research and Clinical Center for Infertility, Shahid Sadoughi University of Medical Sciences	Not reported	Attempted; No response.
Karimzade et al., (2010) [13], Iran, single center, Archives of Obstetrics and Gynaecology	1 June,2008- 1 January, 2009	156 (endometrial scratching: 77, control: 79)	Computer generated randomization method	Not reported	Not reported	Not reported	Implantation (gestational sacs on U/S)	Not reported	Yes (retrospectively)	Attempted; No response
Narvekar et al., (2010) [14], India, single center, Journal of Human Reproductive Sciences	May 2007- July 2008	100 (endometrial scratching: 49, control: 51)	Computer-generated random numbers	Third- party sealed consecutively numbered opaque envelopes	Non-blind	Not reported	Live birth (definition not reported)	Not reported	Not reported	Attempted; No response
Safdarian et al., (2011) [15], Iran, single center, Iranian Journal of Reproductive Medicine	July 2008- March 2009	100 (endometrial scratching: 50, control: 50)	Computerized randomization	Not reported	Not reported	Not reported	Implantation (definition not reported)	Infertility Center, Shariati Hospital, Tehran University of Medical Sciences, Tehran, Iran	Yes (retrospectively)	Attempted; No response
Baum et al., (2012) [16], Israel, single center, Gynecological Endocrinology	July 2006- June 2009	36 (endometrial scratching: 18, control: 18)	Table of random numbers	Not reported	Single-blind (patients did not know in which group they were)	Not reported	Implantation (definition not reported) Clinical pregnancy (intrauterine gestational sac with embryonic pole on U/S)	Not reported	Not reported	Attempted; No response
Inal et al., (2012) [17], Turkey, single center, European Journal of General Medicine	January 2008- March 2009	100 (endometrial scratching: 50, control: 50)	Computer-generated random numbers	Not reported	Not reported	Not reported	Live birth (Definition not reported)	None	Not reported	Attempted; No response
Shohayeb et al., (2012) [18], Egypt and Saudi Arabia, double center, European Journal of Obstetrics & Gynecology and Reproductive Biology	Not reported	210 (endometrial scratching: 105, control: 105)	Random number tables	Third- party closed sealed envelopes	Not reported	Not reported	Implantation (attachment of the embryo to the endometrium during a specific period which is called the window of implantation) Clinical pregnancy (gestational sac with embryonic cardiac activity) Abortion (definition not reported) Live birth (definition not reported)	Not reported	Not reported	Attempted; No response
Nastri et al., (2013) [19], Brazil, single center, Ultrasound Obstetrics Gynecology	June 2010- March 2012	158 (endometrial scratching: 79, control: 79)	Computer generated random sequence of numbers in blocks of 30 (each block having 15 numbers assigned to intervention and 15 to control)	Third- party sealed consecutively numbered opaque envelopes Assigned as the participant entered the study; opened just before the procedure	Double-blind	Yes	Clinical pregnancy (at least one fetus with cardiac activity per allocated woman)	Brazilian official government research foundations: CNPq and CAPES	Yes	Attempted; No response
Guven et al., (2014) [20], Turkey, single center, European Journal of obstetrics and gynecology and reproductive biology	September 2010- April 2011	124 (endometrial scratching: 62, control: 62)	Not reported	Sealed envelopes	Not reported	Not reported	Clinical pregnancy (intrauterine gestational sac with embryonic cardiac activity on TVS, 4 weeks after ET)	Not reported	Not reported	Attempted; No response
Yeung et al., (2014) [21], Hong Kong, single center, Human Reproduction	March 2011- August 2013	300 (endometrial scratching: 150, control: 150)	Randomization in a 1: 1 ratio according to a computer-generated randomization list with blocks of 10 in sealed envelopes	Third- party sealed envelopes	Non-blind	Yes	Ongoing pregnancy (at least one sac with embryonic cardiac activity on U/S beyond 20 weeks of gestation)	Small Project Funding of the Committee on Research and Conference Grants, University of Hong Kong	Yes	No
Gibreel et al., (2015) [22], Egypt, multicenter, Gynecology Endocrinology	Not reported	387 (endometrial scratching: 193, control: 194)	Computer-generated tables of random numbers	Opaque sealed envelopes (on the day of start of pituitary downregulation)	Single-blind (patients did not know in which group they were)	Yes	Live birth (delivery of one or more living fetuses after 24 weeks of gestation)	Not reported	Yes	Attempted; No response.
Singh et al., (2015) [23], India single center, Journal of Human Reproductive Sciences	April 2013- July 2014	60 (endometrial scratching: 30, control: 30)	Random allocation software	Not reported	Non-blind	Not reported	Implantation (gestational sac on TVS)	None	Not reported	Attempted; No response
Xu et al., (2015) [24], China, single center, Reproductive BioMedicine Online	July 2012- July 2013	79 (endometrial scratching+G-CSF: 13, G-CSF: 14, control: 52)	Randomized number table	Not reported	Not reported	Not reported	Endometrial thickness Clinical pregnancy (gestational sac containing yolk sac at TVS, including ectopic pregnancy) Live birth (definition not reported) Implantation (gestational sac on TVS 4 weeks after ET)	Not reported	Not reported	Attempted; No response
Zhang et al., (2015) [25], China, double center, Chinese Journal of Integrative Medicine	August 2009- March 2012	168 (endometrial scratching: 55, Chinese medicine: 56, control: 57)	Randomization with computer-generated list (concealed to the physician but not to the study nurse)	Not reported	Not reported	Not reported	Biochemical pregnancy (positive serum β-hCG level on day 14 after FET) Clinical pregnancy (intrauterine gestational sac with a cardiac activity 3 weeks after a positive β-hCG test)	Shanghai Municipal Health Bureau Foundation of Chinese Traditional Medicine	Not reported	Attempted; No response
Aflatoonian et al., (2016) [26], Iran, single center, International Journal of Reproductive Biomedicine	March 2015- January 2016	100 (endometrial scratching: 50, control: 50)	Computer-generated randomization table	Not reported	Non-blind	Yes	Implantation (gestational sacs on TVS) Clinical pregnancy (gestational sac and embryonic cardiac activity on TVS 5 weeks after ET)	Research and Clinical Center for Infertility, Shahid Sadoughi University of Medical Sciences, Yazd, Iran	Yes (retrospectively)	Attempted; No response
Shahrokh-Tehraninejad et al., (2016) [27], Iran, single center, Journal of Family and Reproductive Health	January 2013- December 2014	120 (endometrial scratching: 60, control: 60)	Manual randomization (drawing a piece of printed paper from a plastic bag)	Not reported	Not reported	Not reported	Clinical pregnancy (intrauterine gestational sac on TVS during week 5 after FET)	Not reported	Yes (retrospectively)	Attempted; No response
Zygula et al., (2016) [28], Poland, European Journal of Obstetrics & Gynecology and Reproductive Biology	Not reported	120 (endometrial scratching: 59, control: 61)	Not reported	Not reported	Not reported	Not reported	Clinical pregnancy (definition not reported)	Not reported	Not reported	Attempted; No response
Liu et al., (2017) [29], China, single center, Reproductive Biology Endocrinology	February 2012- November 2014	142 (endometrial injury in proliferative phase: 38, endometrial injury in luteal phase: 32, control in proliferative phase: 36, control in luteal phase: 36)	Table of random numbers	Not reported	Single-blind (patients did not know in which group they were)	Not reported	Implantation (intrauterine gestational sac on U/S)	National Natural Science Foundation of China, Beijing Natural Science Foundation Project and Project Training High-Level Medical Technical Personnel in the Health System in Beijing	Yes (retrospectively)	Attempted; No response
Mak et al., (2017) [30], Hong Kong, single center, Reproductive Biomedicine Online	March 2013- April 2016	229 (endometrial scratching: 115, control: 114)	Computer-generated random numbers	Third party opaque sealed envelopes	Double-blind	Yes	Pregnancy (Positive urine pregnancy test)	Not reported	Yes	No
Tk et al., (2017) [31], India, single center, European Journal of Obstetrics, Gynecology and Reproductive Biology	April 2008- April 2015	111 (endometrial scratching: 55, control: 56)	Computer generated sequence generated in blocks of 10	Consecutively numbered sealed opaque envelopes	Non-blind	Yes	Clinical pregnancy (gestational sac on U/S)	None	Yes (retrospectively)	Attempted; No response
Maged et al., (2018) [32], Egypt, single center, International Journal of Gynecology and Obstetrics	January 1, 2016- March 31, 2017	300 (endometrial scratching: 150, control: 150)	Automated web-based randomization system	Sealed envelopes	Non-blind	Yes	Clinical pregnancy (embryonic cardiac activity within a gestational sac on U/S 4 weeks after ET) Implantation (gestational sacs on U/S 14 days after ET)	Not reported	Not reported	Yes
Pecorino et al., (2018) [33], Italy, single center, Italian Journal of Gynaecology and Obstetrics	Not reported	80 (endometrial scratching: 40, control: 40)	Not reported	Not reported	Non-blind	Not reported	Clinical pregnancy (intrauterine sac with embryonic cardiac activity on U/S) Implantation (definition not reported)	Not reported	Not reported	Attempted; No response
Sherif et al., (2018) [34], Egypt, single center, Middle East Fertility Society Journal	Not reported	60 (endometrial scratching: 30, control: 30)	Computer-generated randomization table (Research Randomizer Version 4.0 software) in a 1: 1 ratio	Not reported	Not reported	Yes	Pregnancy (definition not reported)	Not reported	Yes	Attempted; No response
Eskew et al., (2019) [35], USA, single center, Journal of Assisted Reproduction and Genetics	September 2013- July 2017	100 (endometrial scratching: 53, control: 47)	Computer-generated block randomization	Consecutively numbered sealed opaque envelopes	Double-blind	Yes	Clinical pregnancy Live birth Miscarriage (definitions not reported)	5T32HD055172-09 and UL1 TR002345	Not reported	Attempted; No response
Frantz et al., (2019) [36], France, multicenter, Human Reproduction	February 2010- July 2014	191 (endometrial scratching: 98, control: 93)	Randomization sequence was generated using SAS Software and was stratified by center with a 1: 1 allocation using random block sizes of 4 and 6	Allocation using random block sizes of 4 and 6	Non-blind	Yes	Clinical pregnancy (at least one intrauterine gestational sac with embryonic cardiac activity)	Ministère de la Santé Français	Yes	Yes
Gurgan et al., (2019) [37], Tuukey, single center, Reproductive Biomedicine Online	February 2015- October 2017	305 (endometrial scratching: 153, control: 152)	Computer-generated random number sequence (1: 1 simple randomization)	Not reported	Not reported	No	Clinical pregnancy (at least one gestational sac with embryonic cardiac activity on U/S) Live birth (definition not reported) Implantation (gestational sacs on U/S)	Not reported	Yes, retrospectively	Attempted; No response
Hilton et al., (2019) [38], Canada, multicenter, Archives of Gynecology and Obstetrics	May 2013- May 2015	51 (endometrial scratching: 25, control: 26)	SAS System for Windows- generated numbers accessed electronically (1: 1 ration, stratification by the study center)	Web-based randomization system	Non-blind	Yes	Clinical pregnancy (documented embryonic cardiac activity 5 weeks after implantation)	Ferring Inc.,Canada	Yes	Yes
Lensen et al., (2019) [5, 6], (New Zeeland, UK, Belgium, Sweden), multicenter, New England Journal of Medicine	June 2014- June 2017	1364 (endometrial scratching: 690, control: 674)	Block randomization of two different sizes between 6 and 16 repeating in random order (1: 1 ratio, stratification according to recruiting site and to whether a fresh-ET or frozen-ET)	Block randomization of two different sizes between 6 and 16 repeating in random order	Non-blind	Yes	Live birth (Definition not reported)	University of Auckland and others	Yes	Attempted; No response
Olesen et al., (2019) [39], Denmark, multicenter, Fertility Sterility	February 2014- December 2017	304 (endometrial scratching: 151, control: 153)	Randomization into blocks of 10 for each participating clinic (1: 1 ratio, according to an Internet-based randomization list)	Consecutively numbered opaque sealed envelopes	Non-blind	Yes	Clinical pregnancy (Definition not reported)	Health Research Fund of the Central Denmark	Yes	Attempted; No response
Berntsen et al., (2020) [40], Denmark, double center, European Journal of Obstetrics and Gynecology and Reproductive Biology	2013- 2018	229 (endometrial scratching: 122, control: 107)	Third-party computer randomization (simple 1: 1 randomization, without using block randomization or stratification)	Not reported	Non-blind	Yes	Positive pregnancy test (serum β-hCG>10 IU/l on day 13–15 after ET)	Department of Gynaecology and Obstetrics at Copenhagen University Hospital Hvidovre	Yes	Attempted; No response
Izquierdo Rodriguez et al., (2020) [41], Spain, single center, Reproductive Sciences	January 2017-October 2018 (follow-up until October 2019)	352 (endometrial scratching: 176, control: 176)	Simple randomization by web-based program	Not reported	Non-blind	Yes	Clinical pregnancy per ET (intrauterine gestational sac on TVS at approximately 6 weeks of gestation)	ProcreaTec Fertility Center	Yes	Attempted; No response
Mackens et al. (2020) [42], Belgium, single center, Human Reproduction	3 April 2014- 8 October 2017	200 (endometrial scratching:100, control:100)	Computer-generated randomization list	Sequentially numbered opaque sealed envelopes	Not reported	Yes	Clinical pregnancy (gestational sac on TVS at 7 weeks of gestation)	Fonds Wetenschappelijk Onderzoek’ (FWO, Flanders, Belgium)	Yes	Yes
Tang et al., (2020) [43], China, single center, Journal of Obstetrics and Gynaecology Research	October 2017- February 2018	220 (endometrial scratching: 110, control: 110)	Manual randomization (sealed envelopes) (details on how the randomization list was generated were not provided)	Sealed envelopes	Non-blind	Yes	Clinical pregnancy (gestational sac on TVS approximately 5 weeks after ET) Live birth (deliveries that resulted in a live born after ET) Implantation (gestational sacs on TVS)	Funded by HeFei Municipal Health Planning Commission, Key Research and Development Project of AnHui Province, Key Talents of Maternal and Child Health in Jiangsu Province and Science Technology Innovation Project of Suzhou	Yes	No
Van Hoogenhuijze et al., (2020) [8, 9], Netherlands, multicenter, Human reproduction	January 2016- July 2018	946 (endometrial scratching: 472, control :474)	Randomization 1: 1 centrally located, non-center-stratified by a web-based programme (ALEA Clinical B.V.) using randomly permuted blocks with block size varying randomly between two and four	Randomly permuted blocks with block size varying randomly between two and four	Non-blind	Yes	Live birth (delivery of at least one live fetus after 24 weeks of gestation)	Dutch organisation for funding of healthcare research ZonMW. The sponsor of the SCRaTCH study was the University Medical Centre Utrecht (UMCU)	Yes	Yes
Metwally et al., (2021) [44], UK, multicenter, Human Reproduction	4^th^ July,2016- 24^th^ October 2018 (follow-up until 24^th^ October, 2019)	1048 (endometrial scratching: 523, control: 525	Randomization sequence generated by the trial statistician using a computer via a web-based system. 1: 1 stratified block randomization was used, with randomly permuted masked blocks of sizes 2, 4, and 6 stratified by site and planned IVF/ICSI (antagonist or long)	Web-based randomization system with restricted access rights that concealed allocation	Non-blind	Yes	Live birth (live birth beyond the 24th week of pregnancy)	National Institute for Health Research	Yes	No
Zahiri et al., (2021) [45], Iran, single center, Galen Medical Journal	Not reported	228 (endometrial scratching: 114, control: 114)	Not reported	Not reported	Not reported	No	Fetal heart activity (assessed via U/S) Multiple pregnancies (assessed via U/S) Abortion (loss of gestational products before the 12^th^ week of gestation)	Vice-Chancellorship of Research and Technology, Guilan University of Medical Sciences	Yes	Attempted; No response
Izquierdo et al., (2022) [46], Spain, single center, Journal of Gynecology Obstetrics and Human Reproduction	January 2017- October 2018 (follow-up until October 2019)	352 (endometrial scratching: 176, control: 176)	Simple randomization by web-based program	Not reported	Non-blind	Yes	Live birth (birth of a living baby beyond the 24th week of pregnancy)	ProcreaTec Fertility Center; Analysis of cumulative Live birth rates did not receive specific funding	Yes	Attempted; No response
Noori et al., (2022) [47], Iran, single center, Journal of Obstetrics and Gynaecology	May 2019- December 2019	100 (endometrial scratching: 50, control: 50)	Sealed envelopes were used as the means of randomization for allocating them into the study groups (details on how the randomization list was generated were not provided)	Sealed envelopes	Not reported	Yes	Chemical pregnancy (β-hCG positive test) Clinical pregnancy (at least 1 intrauterine gestational sac with embryonic cardiac activity)	Research Department of Zahedan University of Medical Sciences	Yes (retrospectively)	Attempted; No response
Turktekin et al., (2022) [48], Turkey, single center, Annals of Clinical and Analytical Medicine	2019-2020	60 (endometrial scratching: 30, control: 30)	Not reported	Not reported	Not reported	Not reported	Clinical pregnancy rate (evidence of a gestational sac, confirmed by U/S at the 4th week of transfer) Live birth (delivery of a live fetus after 24 completed weeks of gestational age) Serum β-hCG levels (measured in all patients on the 12th day of embryo transfer) Miscarriage (loss of fetus before 20 weeks of gestation)	None	Not reported	Attempted; No response

*ET* Embryo transfer, *FET* Frozen-thawed embryo transfer, *hCG* Human chorionic gonadotrophin, *TVS* Transvaginal scan, *U/* Ultrasonographic scan

**Table 2:** Clinical characteristics of included studies

				**Endometrial Scratching group**			**Control group**			
**Study**	**Inclusion criteria**	**Exclusion criteria**	**Type of embryo transfer**	**Method of endometrial injury**	**Timing of intervention**	**Instrument used**	**Control/ Type of intervention**	**Timing of intervention**	**Other interventions**	**Definitions of Pregnancy outcomes**
Karim Zadeh et al., (2008) [11]	Women that have undergone ART treatment cycles with at least 2 implantation failures	Not reported	Fresh ET	Single endometrial biopsy	Luteal phase of cycle preceding IVF	Novak curette	No intervention reported	NA	Not reported	Not reported
Karimzadeh et al., (2009) [12]	Women 20-40 years old with RIF: 2-6 failed IVF-ET cycles and the transfer of >10 high grade embryos per patient without the achievement of clinical pregnancy	1. Blood diseases 2. Poor responders in previous cycles defined as day 3 FSG:3 IU/ml or less than 4 follicles on the day of triggering 3. Uterine malformation 4. Endometrioma 5. Hydrosalpinx (U/S)	Fresh ET	Single endometrial biopsy	Day 21-26 of spontaneous cycle preceding IVF	Pipelle biopsy catheter (Pipelle de Cornier, Prodimed, Neuily-en-Thelle, France)	No intervention reported	NA	Not reported	Chemical pregnancy: measuring serum β-hCG level 14 days after ET (no threshold reported) Clinical pregnancy: intrauterine gestational sac with embryonic cardiac activity on TVS (timing of the assessment not reported)
Karimzade et al., (2010) [13]	1. Women< 38 years 2. BMI: >19 or <30 kg/m^2^ 3. Day 3 FSH<12 IU/L 4. Triple layer endometrium with diameter more than 8 mm on the day of hCG administration 5. Normal ovarian response to COH defined as E2 on the day of hCG administration between 500 and 3,000 pg/mL and number of retrieved oocytes between 4 and 14	1. Uterine anomaly or pathology such as myoma and endometrial polyp 2. Endometriomas with a diameter >3 cm 3. Hydrosalpinges (TVS)	Fresh ET	Single endometrial biopsy; 2 small biopsies obtained from anterior and posterior walls of uterus with a Novak curette	Oocyte retrieval day (34–36 h after hCG administration)	Novak curette	No intervention reported	NA	ES and C: Prophylactic antibiotics (cefazolin 1 g IV)	Clinical pregnancy: gestational sac with embryonic cardiac activity (timing not reported) Ongoing pregnancy: pregnancy proceeding beyond 12 weeks of gestation
Narvekar et al., (2010) [14]	Women≤37 years old with at least 1 previous failed fresh autologous IVF-ET/ICSI cycle with at least 4 good-quality embryos (grade I and II)	1. Previous endometrial tuberculosis (including those treated with antituberculous treatment) 2. Intramural fibroids distorting the endometrial cavity/ submucous myomas/ Asherman’s syndrome 3. Hydrosalpinx	Fresh ET	Double endometrial biopsy; Pipelle introduced through the cervix, piston withdrawn, 360 degrees rotation, 4 up and down movements	Day of hysteroscopy 7-10 of cycle preceding IVF-ET Day 24-25 of cycle preceding IVF-ET	Pipelle biopsy catheter (Pipelle; Gynetics Medical Products, Hamont-Achel, Belgium)	No intervention reported	NA	ES and C: Doxycyclin 100 mg twice daily for 7 days after both the procedures Nonhormonal contraception in the cycle preceding IVF-ET ES: Diclofenac 50mg prior biopsy	Clinical pregnancy: embryonic cardiac activity in US (timing of assessment not reported)
Safdarian et al., (2011) [15]	Women 20-39 years old (Patients with PCO not excluded)	1. FSH>11 IU/L 2. Endometriosis 3. Hypothalamic amenorrhea 4. Azoospermic male	Fresh ET	Single endometrial biopsy	Day 21 of cycle preceding IVF-ET (use of contraceptive pill)	Pipelle biopsy catheter (Piplle-de Cornier, Prodimed, Neuilly-en-Thelle, France)	No intervention reported	NA	ES: Contraceptive pill before the IVF-ET treatment	Not reported
Baum et al., (2012) [16]	1. Women 18-41 years old 2. RIF: ≥3 failed IVF-ET cycles of good morphology embryos to a normal uterus, with good ovarian response in previous cycles 3. Women scheduled for IVF with fresh embryo transfer on the next cycle	1. Uterine malformation 2. Endometrioma 3. Hydrosalpinx (U/S)	Fresh ET	Double endometrial biopsy	Day 9–12 and 21–24 of the spontaneous cycle preceding IVF	Pipelle biopsy catheter (Pipelle de Cornier; Prodimed, Neuillyen-Thelle, France)	Sham procedure; Biopsy catheter into the cervix without scraping	Day 9–12 and 21–24 of the spontaneous cycle preceding IVF	Not reported	Clinical pregnancy: intrauterine gestational sac with embryonic pole on U/S (timing of assessment not reported)
Inal et al., (2012) [17]	Good responders to hormonal stimulation, who failed to conceive during ≥1 cycles of IVF/ET	1. Hydrosalpinx 2. Thrombophilia 3. Submucous myoma 4. Other factors with negative impact on implantation	Fresh ET	Double endometrial biopsy; Pipelle introduced through the cervix, piston withdrawn, 3-4 times rotation in uterine cavity	Two biopsies with one-week interval during the luteal phase of the cycle preceding IVF	Pipelle biopsy catheter (Pipelle; de Cornier, Prodimed, Neuilly-en-Thelle, France)	No intervention reported	NA	ES: Antibiotics administered	Positive test: serum β-hCG>10 microIU/ml measured 12-14 days after the ET Clinical pregnancy: embryonic cardiac activity on US (timing of assessment not reported) Ongoing pregnancy: pregnancy reaching 12th gestational week
Shohayeb et al., (2012) [18]	1. Normal thin endometrium (<5 mm) on day 4 of menstruation 2. Women<39 years old 3. ≥2 previous failed IVF/ICSI cycles (RIF: Failure to achieve pregnancy after 2-6 ICSI cycles with the transfer of more than 10 high grade embryos)	1. Submucous myoma distorting the endometrial cavity 2. Endometrial polyp distorting the endometrial cavity 3. Asherman's syndrome 4. Septate/ Bicornuate uterus (TVS or hysterosalpingography)	Fresh ET	Hysteroscopy and single endometrial biopsy regimen (S-EBR)	Day 4–7 of the cycle preceding IVF-ET	Novak curette	Sham procedure; Hysteroscopy without endometrial scraping	Day 4–7 of the cycle preceding IVF-ET	Not reported	Clinical pregnancy: intrauterine gestational sac with embryonic cardiac activity (timing of assessment not reported)
Nastri et al., (2013) [19]	Women<38 years old who would be submitted to COS, oocyte retrieval and ET	Not reported	Fresh ET	Hysteroscopy and single endometrial biopsy; Pipelle introduced through the cervix, piston drawn back until self-locked, back and forth movements (2-4 cm) while rotating the sampler over the whole uterine cavity for 30 s. If pipelle suction orifice clogged before 30-s period, procedure restarted with another pipelle for another 30 s	7–14 days before starting OS	Pipelle biopsy catheter (Pipelle de Cornier, Laboratoires Prodimed, Neully-En-Thelle, France)	Sham procedure; Drying the cervix with gauze for 30 s	7–14 days before starting OS	ES and C: Oral contraceptives (ethinyl estradiol 30 mcg+levonorgestrel 150 mcg) since last menstruation, for at least 10 days before the appointment	Clinical pregnancy: at least one fetus with cardiac activity (timing of assessment not reported) Live birth: at least one liveborn baby Multiple pregnancy: presence of more than one fetus with cardiac activity Spontaneous miscarriage: loss of a clinical pregnancy before 20 completed weeks of gestation per clinical pregnancy
Guven et al., (2014) [20]	1. Women<35 years old 2. No previous IVF cycles and primary infertility 3. Normoresponders (antral follicle count of 5 to 10 in one ovary in early follicular phase) 4. Grade I or II embryos for transfer 5. Agreement to undergo endometrial biopsy during the COH cycle	1. Endocrinopathies (including diabetes mellitus, hyperprolactinemia, Cushing’s disease and congenital adrenal hyperplasia) 2. Systemic diseases 3. Collagen disorders 4. Hypercholesterolaemia 5. Sickle cell anaemia 6. History of neoplasm 7. High risk for/ history of OHSS 8. Concurrent medication 9. Failure of follicle retrieval 10. Severe male infertility requiring TESA 11. Mullerian tract anomalies 12. History of endometrial instrumentation or surgery within 1 month of the study 1 3. Uterine factors (fibroids, polyps, adhesions) 14. Lack of agreement to undergo ES during the COH cycle	Fresh ET	Single endometrial biopsy; Scratching of anterior and posterior portions of the uterine cavity	Day 3 of the menstrual cycle following downregulation with leuprolide acetate	Biopsy catheter (Gynetics 4164 Probet Pipella; HD Aksu Medical, Ankara, Turkey)	No intervention reported	NA	None	Clinical pregnancy: gestational sac with embryonic cardiac activity on U/S, 4 weeks after ET
Yeung et al., (2014) [21]	1. Subfertile women indicated for IVF treatment 2. Normal uterine cavity demonstrated by saline infusion sonogram or hysteroscopy	1. Endometrial polyp distorting the uterine cavity 2. Fibroid distorting the uterine cavity 3. Hydrosalpinx 4. IVF for PGD 5. Use of donor oocytes	Fresh ET	Hysteroscopy and single endometrial biopsy; Pipelle introduced through the cervix up to the uterine, piston withdrawn, back and forth movements between the fundus and internal os at least 3-4 times	7 days after the LH surge in ovulatory women/ Day 21 of cycle immediately preceding IVF (anovulatory women)	Pipelle biopsy catheter (Pipelle de Cornier, Laboratoire C.C.D., France)	No intervention reported	NA	Not reported	Ongoing pregnancy: at least one embryonic cardiac activity on U/S beyond 20 weeks of gestation Clinical pregnancy: at least one gestational sac on U/S at 6 weeks of gestation Miscarriage: number of miscarriages before 20 weeks of gestation Multiple pregnancy: more than one gestational sac detected on U/S at 6 weeks of gestation
Gibreel et al., (2015) [22]	Women aged<40 years with at least 1 previous failed IVF cycle	1. Poor responders after previous IVF treatment 2. Endocrinopathy 3. Tubal disconnection for hydrosalpinx 4. History of endometrial curettage within 3 months of the study 5. Fibroids and other factors distorting the endometrial cavity (e.g., polyps or adhesions)	Fresh ET	Double endometrial biopsy; Pipelle introduced through the cervix up to the uterine fundus, then withdrawn for 1 cm, piston drawn back until self-locked. 2-3 back-and-forth movements	Day 21 and day 23-24 of the cycle preceding IVF	Pipelle biopsy catheter (Laboratoires Prodimed, Neully-En-Thelle, France)	Sham procedure; Introduction of a sound through the cervix, stopped just before crossing the internal OS	Day 21 and day 23-24 of the cycle preceding IVF	ES and C: Combined oral contraceptive pills from day 5 of the cycle preceding IVF	Live birth: delivery of one or more living fetuses after 24 weeks of gestation Clinical pregnancy: gestational sac with embryonic cardiac activity on U/S 4 weeks after ET
Singh et al., (2015) [23]	1. Women<35 years old with >1 previous failed IVF attempts 2. Good ovarian reserve (AFC>8, AMH: 2–6 ng/ml, FSH<8 IU/L) 3. No uterine manipulation within last 3 months (e.g., hysteroscopy, myomectomy) 4. Willingness to participate in the trial	1. Women>35years old with confounding factors (e.g., poor ovarian reserve) 2. Grade III and IV endometriosis 3. History of septal resection or adhesiolysis 4. Uterine malformation 5. Other possible causes for failure of implantation (e.g., diabetes mellitus, hypertension, autoimmune diseases)	Fresh ET	Single endometrial injury; Karman’s cannula introduced through the cervix, anterior and posterior walls of endometrium scratched gently (4 mm)	Day 14-21 of cycle preceding IVF-ET	Karman's cannula	No intervention reported	NA	ES and C: Ciprofloxacin 500mg per os for 5 days	Not reported
Xu et al., (2015) [24]	1. Women<40 years old 2. FSH<10 IU/L 3. Failure of TEM to reach 7 mm by regular methods 4. No signs of submucosal uterine myoma, uterine malformations, endometrial polyps, or obvious IUA by TVS or diagnostic hysteroscopy 5. No signs of other diseases which could have affected endometrial growth 6. No contraindications for G-CSF treatment (e.g., chronic neutropenia, sickle cell disease, renal disease and history of malignancy)	Not reported	Frozen ET	Intrauterine G-CSF+single endometrial biopsy; Biopsy catheter introduced through the cervix until uterine fundus reached, piston withdrawn and the endometrium lightly scratched 1-2 times up and down on every wall of the uterine cavity, with abdominal US guidance. 300 g of G-CSF (100 g/0.6 ml) were injected into the cavity with the help of a 2-ml syringe and an embryo transfer catheter	On the day that one follicle became dominant-diameter: 12x12 mm	Endometrial biopsy catheter (Gynetics Medical Products N.V., Lommel, Belgium)	Intrauterine G-CSF; Under abdominal US guidance, 300 g of G-CSF (100 g/0.6 ml) were injected into the cavity with the help of a 2-ml syringe and an embryo transfer catheter	On the day that one follicle became dominant-diameter: 12x12 mm	ES: Intrauterine G-CSF after endometrial injury	Clinical pregnancy: gestational sac containing yolk sac on TVS, including ectopic pregnancy (timing of assessment not reported) Spontaneous abortion: loss of a clinical pregnancy of less than 20 weeks of gestation Implantation: gestational sacs on TVS, at least 4 weeks after ET
Zhang et al., 2015 [25]	1. RIF: 3 or more implantation failures in previous IVF/ICSI cycles 2. High-quality embryos subjected to cryopreservation by vitrification and still in good condition after being thawed	Not reported	Frozen ET	Hysteroscopy and single endometrial biopsy	Not reported	Digital camera (Tricam SLII, Germany, Carl Stortz, Tuttlingen, Germany) (Catheter used not reported)	No intervention reported	NA	ES: Hysteroscopy	Chemical pregnancy: β-hCG positive test (threshold not reported) Clinical pregnancy: At least 1 intrauterine gestational sac with embryonic cardiac activity (timing of assessment not reported)
Aflatoonian et al., (2016) [26]	1. Women <40 years old indicated for FET treatment 2. 1 or more frozen embryo(s) 3. Normal uterine cavity (TVS)	1. History of endocrinopathies (hypothyroidism, diabetes mellitus) 2.Intrauterine abnormality (uterine polyp, submucosal fibroma, intrauterine adhesion) 3. Severe endometriosis (laparoscopy) 4. Endometrioma (U/S)	Frozen ET	Single endometrial biopsy; Pipelle introduced through the cervix up to uterine fundus, piston drawn back, sheath rotation and 2-3 back and forth movements	Day 21-23 of cycle preceding ET	Pipelle biopsy catheter (Endobiops, Prince Medical France)	No intervention reported	NA	Not reported	Chemical pregnancy: positive serum β-hCG test 14 days after ET Clinical pregnancy: gestational sac and embryonic cardiac activity on U/S 5 weeks after ET Ongoing pregnancy: embryonic cardiac activity on U/S beyond 12 weeks of gestation Miscarriage rate: loss of pregnancy <20 weeks of gestation
Shahrokh-Tehraninejad et al., (2016) [27]	1. Women<40 years old, 2. RIF: ≥2 previous failed IVF/ICSI cycles 3. ≥4 embryos with good quality (grade I) 4. Normal uterus in hysterosalpingography, sonography, hystrosonography or hysteroscopy 5. ≥7mm endometrium thickness at suppository progesterone administration day	1. Submucousal, intramural and subserousal myoma>5 cm 2. Endometrioma≥3 cm 3. Hydrosalpinx 4. Bilateral obstruction of tube 5. <3-4 embryos 6. Endometrial tuberculosis or history of tuberculosis treatment 7. Asherman’s syndrome 8. BMI>30 kg/m^2^ 9. Active vaginal or cervical infection 10. Systemic diseases (e.g., diabetes or systemic lupus erythematous)	Frozen ET	Single endometrial biopsy; Evaluation for LEI, endometrial injury in all 4 uterine walls by up and down movements of pipelle catheter in the uterine cavity	Day 21 of cycle preceding ET	Pipelle biopsy catheter	No intervention reported	NA	Not reported	Clinical pregnancy: intrauterine gestational sac on TVS during week 5 after ET
Zygula et al., (2016) [28]	1. Women< 40 years old with previous IVF failure	Not reported	Fresh ET	Single endometrial biopsy	Day 21 of cycle preceding IVF	Pipelle biopsy catheter	No intervention reported	NA	Not reported	Not reported
Liu et al., (2017) [29]	1. Infertile women indicated for IVF treatment 2. Women≤40 years old 3. Normal uterine cavity demonstrated by saline infusionsonogram 4. bFSH<12 IU/L	1. Factors distorting the endometrial cavity (polyp, fibroid) 2. Hydrosalpinx 3. Endometriosis	Fresh ET	Single endometrial injury; Pipelle catheter introduced through the cervix up to the uterine fundus, piston drawn back, sheath rotation and back and forth movements within the uterine cavity	Proliferative phase group: day 10–12 of cycle preceding IVF Luteal phase group: 7–9 days after ovulation	Pipelle biopsy catheter (Shanghai Jiabao Medical Healthy Science Company, Shanghai, China)	Sham procedure- No endometrial scratching	Proliferative phase group: day 10–12 of cycle preceding IVF Luteal phase group: 7–9 days after ovulation	Not reported	Clinical pregnancy: intrauterine gestational sac and embryonic cardiac activity at 6 weeks of gestation Biochemical pregnancy: positive serum β-hCG (threshold not reported)
Mak et al., (2017) [30]	All patients deemed suitable for natural-cycle FET and scheduled for FET cycles using non-donor oocytes, with normal ovulation	Uterine malformation or other pathology (e.g., polyps, endometriomas>4 cm, hydrosalpinx)	Frozen ET	Single endometrial biopsy; Pipette catheter introduced through the cervix, inner part of the device withdrawn, up and down movements approximately 2–3 cm within the uterine cavity. The procedure repeated at least 4 times with 360 degrees device rotation	Mid-luteal phase of cycle preceding ET (FET: 7±1 days after the surge of LH)	Biopsy catheter (Pipette; MedGyn, USA)	Sham procedure; Endocervical manipulation with sterile cotton wool stick inserted 2 cm into the cervical os, moved up and down and rotated 360°	Mid-luteal phase of cycle preceding ET (FET: 7±1 days after the surge of LH)	Not reported	Pregnancy: positive urine pregnancy test Clinical pregnancy: confirmed intrauterine gestational sac Ongoing pregnancy: at least one fetus with cardiac activity beyond 32 weeks of gestation Live birth: at least one live-born infant (minimum weeks of gestation not reported)
Tk et al., (2017) [31]	1. At least 1 previous failed IVF cycle with minimum of 2 good quality embryos (cleavage or blastocyst stage) transferred in an earlier attempt 2. Women≤38 years old 3. BMI≤29 kg/m^2^ 4. FSH<10 IU/L	1. Previous poor response (<3 oocytes retrieved in previous cycle) 2. Endometrial pathology 3. Uterine malformations 4. Severe endometriosis 5. Gross adenomyosis 6. Systemic diseases (e.g., autoimmune disorders)	Fresh ET	Double endometrial biopsy	Biopsy twice within 48h in the luteal phase of cycle preceding COH	Pipelle biopsy catheter	No intervention reported	NA	None	Biochemical pregnancy: β-hCG>5 mIU/ml level on day 18 after oocyte retrieval Clinical pregnancy: intrauterine gestational sac on U/S (timing of assessment not reported) Live birth: delivery of live fetus after 24 weeks of gestation Miscarriage: loss of pregnancy <24 weeks of gestation Multiple pregnancy: more than one gestational sac on early U/S Preterm delivery: delivery between 24 and 37 weeks of gestation
Maged et al., (2018) [32]	1. First ICSI cycle 2. Women< 40 years old 3. Day-3 FSH<10 IU/L 4. Normal serum prolactin 5. No uterine cavity abnormality	1. Endocrinopathies (e.g., abnormal thyroid or adrenal function) 2. Ovarian cysts 3. Hydrosalpinx 4. Polyps 5. Azoospermia 6. ICSI for PGD	Fresh ET	Single endometrial biopsy; Pipelle catheter introduced through the internal os up to uterine fundus, piston withdrawn, sheath rotation and movements 3-4 times between fundus and inner os	Mid-luteal phase of the cycle immediately preceding IVF	Pipelle biopsy catheter (Cooper Surgical, Trumbull, CT, USA)	No intervention reported	NA	Not reported	Clinical pregnancy: embryonic cardiac activity within a gestational sac on U/S 4 weeks after ET Multiple pregnancy: multifetal pregnancy 4 weeks after ET Abortion: spontaneous abortion before 12 weeks of gestation
Pecorino et al., (2018) [33]	1. Women 25-37 years old with primitive or secondary infertility 2. At least 2 previous failed ICSI or FIVET (failed implantation) despite easy transfer and good quality embryos 3. Normal thickness and endometrial U/S pattern, defined as absence of intracavitary disease (fibroids, polyps, etc.), with no anamnestic severe deep endometriosis 4. Good quality of seminal fluid of partner and negative anamnesis for relevant diseases 5. Negative genetic, metabolic and infective evaluation	Not reported	Mixed	Single endometrial biopsy; Pipelle introduced through the cervix up to the uterine fundus, piston drawn back until self-locked, back and forth movements (3-4 cm) and then rotating movements over the whole uterine cavity for 30 s	Day 5-10 of cycle preceding IVF	Pipelle biopsy catheter (pipelle de Cornier® (Laboratoires PRODIMED, Neully-EnThelle, France)	Sham procedure; Embryo-transfer catheter inserted through the cervix in the uterine cavity	Day 5-10 of cycle preceding IVF	Not reported	Clinical pregnancy: intrauterine sac with embryonic cardiac activity on U/S (timing of assessment not reported)
Sherif et al., (2018) [34]	1. Age is between 25-30 years old. 2. BMI between 20 and 30 kg/m^2^ 3. Cause of infertility: tubal causes, ovulatory causes, unexplained causes of infertility	1. Women>30 years old 2. BMI>30 kg/m^2^ 3. Endometriosis 4. Male factor infertility 5. Uterine malformations (U/S or HSG) 6. Previous failed ICSI 7. Hydrosalpinx and pyosalpinx (U/S)	Fresh ET	Single endometrial injury-modified COOK catheter movements on the posterior endometrium 1–2 cm from the fundus under U/S guidance	Day 6 of IVF-ICSI cycle	Modified COOK catheter	No intervention reported	NA	ES and C: Combined Oral Contraceptive from day 2 or day 3 of cycle preceding IVF for 21 days	Not reported
Eskew et al., (2019) [35]	Women 18–43 years old undergoing a fresh or frozen embryo transfer	1. Abnormal endometrial cavity evaluation 2. Third-party reproduction cycles	Mixed	Single endometrial biopsy; Cervix disinfection with an iodine solution, pipelle catheter introduced through the cervix to the fundus, plunger withdrawn, sheath rotation and 3-4 up and down movements, up to 2 passes	Patients OCP: during the last 7 days or up until 1 day after pills were discontinued (cycle preceding IVF-ET) Patients nOCP: Check for LH surge and ES 7–13 days following in the cycle preceding IVF-ET	Pipelle biopsy catheter (Endocell™ Trumbull, CT)	Sham procedure; Cervix disinfection with an iodine solution. Pipelle inserted into the posterior fornix and plunger withdrawn. Up and down movements of pipelle behind the cervix 3-4 times	Patients OCP: during the last 7 days or up until 1 day after pills were discontinued (cycle preceding IVF-ET) Patients nOCP: Check for LH surge and ES 7–13 days following in the cycle preceding IVF-ET	Not reported	Not reported
Frantz et al., (2019) [36]	1. 18–38 years old 2. 1 or no previous failed IVF cycle 3. Primary or secondary infertility 4. Regular menstrual cycles (between 27 and 32 days) 5. FSH ≤2 IU/L	1. Participation to oocyte donation program 2. BMI>35 kg/m^2^ 3. Hydrosalpinx 4. Uterine malformations 5. Fibroids (≥4 and the largest >5 cm) 6. Abnormal gynecological bleeding 7. Active vaginal infection 8. Pre-treatment with estrogen–progesterone or estradiol per os 9. Participation in another medically assisted reproduction study	Fresh ET	Single endometrial biopsy; Suction and rotation with a Pipelle catheter	Day 20-24 of cycle preceding IVF	Pipelle biopsy catheter (Pipelle de Cornier, CCD international, PROMIDED, Neuilly-en Thelle, France)	No intervention reported	NA	Not reported	Clinical pregnancy rate: at least one intrauterine gestational sac with embryonic cardiac activity Ongoing pregnancy: ≥12 weeks of gestation
Gurgan et al., (2019) [37]	1. Women<40 years old 2. RIF: failure to achieve clinical pregnancy after at least 4 good-quality embryos transferred in a minimum of 3 fresh or frozen cycles 3. FSH≤15 IU/L	1. Congenital uterine malformations 2. Asherman's syndrome 3. Myoma or endometrial polyps distorting the endometrial cavity 4. Endometriosis or endometrioma 5. BMI<18.5 or >29.9 kg/m^2^ 6. Endometrial thickness<7 mm in the cycle before ART	Mixed	Office hysteroscopy and single endometrial injury; Under sedation, 5 mm 30° lens supplied with a 5F working channel continuous flow office hysteroscope introduced through the cervix, endometrial injury with scissors first on the fundus by cutting transversally into the endometrium, then 3-4 vertical incisions 0.5 cm apart on the anterior and posterior walls of the uterus, 1-1.5 cm away from the fundus and with 1 cut for each lateral wall	Day 10-12 of cycle preceding IVF	5 mm 30° lens supplied with a 5 F working channel continuous flow office hysteroscope (Bettocchi® Integrated Office Hysteroscope; KARL STORZ, Tuttlingen, Germany), scissors	No intervention reported	NA	None	Clinical pregnancy: at least one intrauterine gestational sac with embryonic cardiac activity on U/S (timing of assessment not reported) Early pregnancy loss: loss of an intrauterine pregnancy within the first trimester Premature birth: birth before 37 weeks of gestation
Hilton et al., (2019) [38]	1. 1 or no previous failed IVF cycle (women on their first or second IVF/ICSI cycle) 2. 18–39 years old 3. BMI 18–35 kg/m^2^ 4. Evaluation of uterine cavity (hysterosalpingogram, sonohysterogram, hysteroscopy) performed in the preceding 24 months 5. Early follicular phase (day 2 or 3) serum FSH evaluated in the preceding 6 months 6. Use of a long GnRH agonist or GnRH antagonist protocol 7. Documented LH surge 9–11 days before enrolment for patients not pretreated with the oral contraceptive pill or use of the OCP for ≥ 10 days at the time of enrollment	1. Previous participation in this study 2. Prior early follicular phase FSH≥12 IU/L 3. Previous poor ovarian response (IVF cycle canceled for poor response or ≤4 oocytes retrieved) 4. IVF for PGD or fertility preservation 5. Endocrinopathies (e.g., diabetes mellitus, uncontrolled thyroid disease) 6. Uterine malformations 7. Untreated hydrosalpinx 8. Contraindications to endometrial biopsy 9. Office hysteroscopy or other uterine procedure planned or performed during the cycle preceding IVF stimulation 10. Use of surgically retrieved sperm in this IVF cycle	Fresh ET	Single endometrial biopsy; No anesthesia. Pipelle catheter introduced through the cervix in the uterine cavity, inner core withdrawn, acquisition of endometrial tissue upon rotation within the cavity until sampling considered adequate for histological assessment by a local pathologist	5–10 days preceding COS	Pipelle biopsy catheter	No intervention reported	NA	Not reported	Clinical pregnancy: documented embryonic cardiac activity 5 weeks after implantation Live birth delivery: deliveries that resulted in at least 1 live birth
Lensen et al., (2019) [5, 6]	Women planning IVF with their own oocytes (stimulated IVF cycle with planned fresh-embryo transfer or frozen-embryo transfer with the use of stored embryos)	1. ET not planned (e.g., fertility preservation or plan to freeze all embryos for storage) 2. Contraindications to pipelle biopsy (e.g., vaginismus) 3. Intrauterine procedures within 3 months before the start of IVF (hysteroscopy, sonohysterography, hysterosalpingography, laparoscopy, surgically managed miscarriage or endometrial biopsy)	Mixed	Single endometrial biopsy; Obtaining of endometrial biopsy sample with pipelle, according to clinic protocols. If inserting the pipelle in the uterus not possible, local anesthetic and cervical dilatation permitted or second attempt scheduled for another day or with a different clinician (or both). (Procedure discontinued at the participant’s request or due to clinician's inability to pass the pipelle)	Between day 3 of the cycle preceding ET and day 3 of the ET cycle	Pipelle biopsy catheter 3 mm in diameter (e.g., Pipelle de Cornier, Laboratoire CCD, France)	No intervention reported	NA	ES: Advice to take pain medication before the procedure	Biochemical pregnancy: positive pregnancy test (timing of assessment not reported) Multiple pregnancy: more than one sac with embryonic cardiac activity by any scan on approximately 6 weeks of gestation Miscarriages: losses of clinical pregnancy before 20 weeks of gestation, excluding ectopic pregnancy Stillbirths: losses of clinical pregnancy at or after 20 weeks of gestation (not including loss of one fetus in multiple pregnancies) Terminations: losses of an intrauterine pregnancy, through intervention by medical, surgical or unspecified means
Olesen et al., (2019) [39]	1. IVF or ICSI patients with 1 or more prior implantation failures, despite top-quality embryo or blastocyst transfer(s) 2. Regular menstrual cycle (28–32 days) 3. 18–40 years old 4. BMI: 18–32 kg/m^2^	1. Congenital uterine malformations 2. Fibroids 3. Polyps 4. Hydrosalpinges 5. Adenomyosis	Fresh ET	Single endometrial biopsy; Patient lying in a lithotomy position and scratching performed once in each quadrant of the endometrium with a pipelle catheter	Day 18–22 of cycle preceding IVF	Pipelle biopsy catheter (Pipelle de Cornier (Laboratoires Prodimed)	No intervention reported	NA	Not reported	Not reported
Berntsen et al., (2020) [40]	Women were 18-40 years old with at least 1 previous failed IVF/ICSI cycle (No criteria for ovarian reserve, no age criteria or other criteria for the male partner or male partner sperm)	1. Freeze-all cycles/ frozen embryo transfers 2. BMI≥35 kg/m^2^ 3. Intrauterine pathology as cause of infertility 4. Significant systemic disorders 5. Ongoing reproductive tract or systemic infection 6. Intrauterine abnormalities diagnosed during trial hysteroscopy 7. Spontaneous pregnancy during the trial	Fresh ET	Office hysteroscopy and single endometrial biopsy; No sedation, unless procedure not possible without local anesthetics. Office hysteroscopy with an evaluation of the uterine cavity and cervical canal the help of hysteroscope and saline as distension media. 1 or 2 biopsies primarily performed on the posterior wall of the uterus (no firm strategy for precise location)	Follicular phase of the cycle preceding IVF	ALPHASCOPETM hysteroscope (GMS40A) 1.9 mm with GYNECARE VERSASCOPETM sheath (GMS805) 3.5 mm (Ethicon, Johnson & Johnson, Livingston, Scotland), 7 F forceps (GIMMI1 GmbH)	No intervention reported	NA	ES: Oral paracetamol 1000 mg and Ibuprofen 400 mg one hour before hysteroscopy	Positive pregnancy test rates: serum β-hCG>10 IU/l on day 13–15 after ET Ongoing pregnancy: at least one intrauterine gestational sac with embryonic cardiac activity at gestational weeks 7-9 Live birth: delivery of a live fetus after 22 weeks of gestation
Izquierdo Rodriguez et al., (2020) [41]	1. 18-50 years old 2. Normal uterine cavity (2D TVS) 3. Patients with endometrial polyps if polypectomy was performed at least 2 months before the treatment cycle	1. Low sperm quality 2. Uterine intervention within 1 month of the study 3. Uterine malformations (fibroids 0–2 FIGO stage, Müllerian malformations, severe adenomyosis) 4. Unilateral or bilateral hydrosalpinx 5. BMI>35 kg/m^2^ 6. Frozen ET	Fresh ET	Single endometrial biopsy; Cervix disinfection with an iodine solution, biopsy catheter inserted through the cervix up to the uterine with abdominal U/S guidance, piston partially removed, back and forth movements and rotation 360 degrees of the catheter in order to scratch the four walls	5 to 10 days before start of period and the endometrial preparation	Pipelle biopsy catheter (Pipelle de Cornier, Laboratoire CCD, France)	No intervention reported	NA	Not reported	Clinical pregnancy: intrauterine gestational sac on TVS at approximately 6 weeks of gestation Pregnancy: positive pregnancy test (serum β-hCG>10 mUI/ml) Ongoing pregnancy: pregnancy continued beyond 12 weeks Early miscarriage: clinical pregnancy lost before 12 weeks Late miscarriage: pregnancy stopped between the 12- 24 weeks of pregnancy Live birth: birth of a live baby beyond the 24 weeks of pregnancy
Mackens et al. (2020) [42]	1. Women 18-40 years old 2. Fresh ART cycle 3. GnRH antagonist down-regulation 4. Signed informed consent	1. Reasons for impaired implantation (e.g., hydrosalpinx, fibroid distorting the endometrial cavity, Asherman’s syndrome, thrombophilia or endometrial tuberculosis) 2. Oocyte donation 3. Frozen ET 4. Embryos planned to undergo embryo biopsy 5. BMI>35 or <18 kg/m^2^ 6. Participation in another study on medically assisted procreation during the same cycle 7. Previous participation in the study 8. Inability to comprehend the investigational nature of the proposed study	Fresh ET	Single endometrial biopsy; Pipelle introduced in the uterus until slight resistance from the fundus, piston withdrawn and 360 degrees device rotation as it was moved up and down 4 times	Day 6-8 of cycle of OS	Pipelle biopsy catheter (Pipelle de Cornier® Laboratoire CCD, France)	No intervention reported	NA	Not reported	Clinical pregnancy: intrauterine gestational sac on TVS at 7 weeks of gestation Cumulative reproductive outcomes: number of biochemical pregnancies, clinical pregnancies, early pregnancy losses and live births, taking into account all conceptions (spontaneous or following ART) within an actively monitored 6-month follow-up period following randomization
Tang et al., (2020) [43]	1. Patients indicated for frozen–thawed ET 2. Serum progesterone level< 1.2 ng/mL on the third day of the menstrual cycle 3. At least 2 or more previous implantation failures 4. Normal morphology of uterine cavity	1. Pelvic surgery history 2. Difficult ET 3. Intrauterine malformations (severe adhesions, polyp, submucosal fibroid) 4.BMI>27 kg/m^2^ 5. Hydrosalpinx 6. Endometriosis 7. Oral contraception drugs recently	Frozen ET	Single endometrial biopsy; Pipelle introduced through the cervix up to the uterine cavity, piston withdrawn and rotation 360 degrees and up and down movements 4 times Sample examined under microscope to evaluate the size and level of the injury and to verify the proliferative state of endometrium	Day 3 of the cycle preceding ET	Pipelle biopsy catheter (Beijing Saipu Jiuzhou Science and Technology Developent Company)	No intervention reported	NA	Not reported	Clinical pregnancy: gestational sac on TVS approximately 5 weeks after ET Biochemical pregnancy: positive β-hCG test 14 days after ET (threshold not reported) Miscarriage rate: loss of pregnancy before 20 weeks
Van Hoogenhuijze et al., (2020) [8, 9]	1. Women with at least 1 full IVF/ ICSI cycle with at least 1 embryo transfer without achieving a clinical pregnancy and planning a new fresh IVF/ICSI cycle 2. Regular indication for IVF/ICSI 3. 18–44 years old 4. Primary or secondary infertility 5. Normal TVS	1. Grade III and IV endometriosis 2. Untreated uni- or bilateral hydrosalpinx 3. Previous endometrial scratching 3. Untreated endocrinopathies 4. Intermenstrual blood loss 5. Previous Caesarean section with niche-formation and intracavitary fluid on US 6. Increased risk of intra-abdominal infection 7. Oocyte donation 8. PGT	Fresh ET	Single endometrial biopsy- performed by suction	Mid-luteal phase. LH surge (+5–8 days), 5–10 days before the expected next menstruation or expected withdrawal bleeding (when taking oral contraceptives)	Biopsy catheter	No intervention reported	NA	Not reported	Clinical pregnancy: intrauterine gestational sac visible on U/S at 6–7 weeks of gestation Ongoing pregnancy: embryonic cardiac activity on U/S at 10 weeks of gestation Live birth: delivery of at least 1 live fetus after 24 weeks of gestation Multiple pregnancy: birth of multiple live fetuses after 24 weeks of gestation Live birth: ongoing pregnancy leading to live birth
Metwally et al., (2021) [44]	1. Women 18–37 years old undergoing their first cycle of IVF, with or without ICSI, expected to be using fresh embryos and a single embryo transfer (SET) 2. Regular ovulatory menstrual cycle defined by clinical judgement or with ovulatory levels of midluteal serum progesterone, normal uterine cavity assessed by TVS at screening 3. No endometrial abnormalities that would require treatment to facilitate pregnancy (e.g., suspected intrauterine adhesions, uterine septae, submucosal fibroids or intramural fibroids >4 cm in diameter) 4. Good ovarian reserve assessed clinically, biochemically (FSH<10 UI/L) and normal follicular phase estradiol levels and/or normal AMH levels or sonographically (antral follicle count) 5. No history of previous radiotherapy or chemotherapy 6. No relevant vaginal/ uterine infections 7. (If randomized) Willingness to use a barrier method of contraception prior to the procedure if necessary	1. Previous trauma to the endometrium (resection of uterine septum, intrauterine adhesions, or recent resection of significant submucous fibroids) 2. BMI≥35 kg/m^2^ 3. Participating in another interventional fertility study 4. Grade IV endometriosis 5. Participants undergoing ultra-long protocols 6. Other endometrial procedures (e.g., endometrial biopsy for the collection of natural killer cells)	Fresh ET	Single endometrial biopsy; Speculum inserted into the vagina, cervix exposed and cleaned. Pipelle sampler or similar device inserted into the cavity of the uterus and plunger withdrawn, sampler rotated and withdrawn 3-4 times so that tissue appeared in the transparent tube	Mid-luteal phase of the cycle preceding IVF (defined as 5–7 days before the expected next period, or 7–9 days after a positive ovulation test)	Pipelle catheter or similar device	No intervention reported	NA	ES: Participants were required to use a barrier method of contraception (if necessary) in the menstrual cycle in which the ES was performed	Implantation: positive serum β-hCG or by a positive urine pregnancy test on approximately day 14 following egg collection Clinical pregnancy: observation of viable intrauterine pregnancy with a positive heart pulsation seen on U/S at/after 8 weeks of gestation Miscarriage: spontaneous pregnancy loss, including pregnancy of unknown location prior to 24 weeks gestation, within the 10.5 month post egg collection follow-up period Ectopic pregnancy: pregnancy outside the normal uterine cavity Multiple birth: the birth of more than one living fetus after completed 24 weeks of gestation Preterm delivery: live birth after 24 weeks and before 37 weeks gestation within the 10.5 month post egg collection follow-up period Stillbirth: delivery of a stillborn fetus showing no signs of life after 24 weeks gestation within the 10.5 month post egg collection follow-up period
Zahiri et al., (2021) [45]	1. History of ICSI failure at least twice 2. Age<40 years old 3. FSH≤12 IU/L 4. Normal ultrasound assessment of uterus (including myometrium and endometrium) 5. Normal HSG or normal laparoscopy assessment	1. Endometrial lesions in hysteroscopy (myoma, polyp, Asherman’s syndrome or Mullerian anomaly) 2. Unavailability of at least 2 embryos of good quality 3. OHSS 4. Serum progesterone >1.5-2 ng/mL 5. Diabetes mellitus, CRF, thyroid disorders, kidney or hepatic diseases 6. Smoking or being exposed to cigarette smoke for at least 3 months prior to the intervention 7. In the case of diagnosing any endometrial lesions, including polyps-fibroma-adhesion or Müllerian anomaly during the patient was excluded from the study	Fresh ET	Hysteroscopy and single endometrial biopsy; Scratching by a curette on four sides of the endometrium (anterior, posterior, and two lateral sides)	Luteal phase of cycle preceding IVF	Curette	Sham procedure-hysteroscopy without intervention	Luteal phase of cycle preceding IVF	Not reported	Abortion: loss of gestational products before 12 weeks of gestation
Izquierdo et al., (2022) [46]	1. 18-50 years old 2. Normal uterine cavity (2D TVS) 3. Patients with endometrial polyps if polypectomy was performed at least 2 months before the treatment cycle	1. Low sperm quality 2. Uterine intervention within 1 month of the study 3. Uterine malformations (fibroids 0–2 FIGO stage, Müllerian malformations, severe adenomyosis) 4. Unilateral or bilateral hydrosalpinx 5. BMI>35 kg/m^2^ 6. Frozen ET	Fresh ET	Single endometrial biopsy; Cervix disinfection with an iodine solution, biopsy catheter inserted through the cervix up to the uterine with abdominal US guidance, piston partially removed, back and forth movements and rotation 360 degrees of the catheter in order to scratch the four walls	5 to 10 days before start of period and the endometrial preparation	Pipelle biopsy catheter (Pipelle de Cornier, Laboratoire CCD, France)	No intervention reported	NA	Not reported	RIF: patients with 2 or more previous failed implantations non-RIF: patients with a maximum of 1 previous failed ET
Noori et al., (2022) [47]	1. Women with primary infertility undergoing their first IVF procedure who had a BMI≤35 kg/m^2^ 2. 20-40 years old 3. Normal uterine cavities in previous HSG or previous hysteroscopy 4. FSH≤12 IU/L	1. Indices of uterine lesions (submucosal uterine leiomyomas or endometrial polyps) 2. History of moderate to severe pelvic endometriosis 3. Diagnosis of moderate to severe male factor infertility based on the WHO indices 4. History of tobacco use or alcohol consumption 5. Previous failed IVFs 6. Lack of proper embryo for transfer	Frozen ET	Single endometrial biopsy	Luteal phase of IVF cycle preceding ET	Pipelle curette	No intervention reported	NA	ES and C: Patients were advised to use Oral Contraceptive Pills from day 3 of cycle following oocyte retrieval or use barrier contraceptive	Chemical pregnancy: β-hCG positive test (threshold and timing of assessment not reported) Clinical pregnancy: At least 1 intrauterine gestational sac with embryonic cardiac activity (timing of assessment not reported)
Turktekin et al., (2022) [48]	1. Women scheduled for total embryo freezing due to the risk of OHSS 2. Patients were diagnosed with PCOS based on the revised Rotterdam criteria, two out of three: (1) oligo and/or anovulation, (2) clinical and/or biochemical hyperandrogenism, and (3) polycystic ovaries determined with U/S	1. Women with Asherman’s syndrome, endometrial polyp, submucous fibroids, uterine septum or other congenital uterine anomalies, hydrosalpinx or endometrioma 2. History of hormonal medication or intrauterine contraception use within the past 12 months 3. History of habitual abortion 4. Endocrine disorders	Frozen ET	Single endometrial biopsy; While the patient still under anesthesia, Pipelle catheter introduced through the cervix up to the uterine fundus, piston withdrawn to create negative pressure, catheter pushed back and forth in the cavity and withdrawn. (Procedure was repeated until most of the cavity was injured)	Day of oocyte retrieval (after the retrieval)	Pipelle biopsy catheter	Sham procedure- Pipelle catheter advanced through the cervix to the fundus and then removed from the cavity, no injury made	Day of oocyte retrieval (after the retrieval)	ES: A single dose of antibiotic prophylaxis was administered to the participants before the procedure	Clinical pregnancy rate: evidence of a gestational sac, confirmed by ultrasound examination at week 4 after ET Live birth: delivery of a live fetus after 24 completed weeks of gestational age Serum β-hCG levels: measured in all patients on the 12th day of embryo transfer (threshold not reported) Miscarriage: loss of fetus before 20 weeks of gestation

*AMH* Anti-mullerian hormone, *C* Control group, *COH* Controlled ovarian hyperstimulation, *ES* Endometrial scratching group, *ET* Embryo Transfer, *FET* Frozen Embryo Transfer, *FSH* Follicle-Stimulating hormone, *HSG* hysterosalpingography, *ICSI* Intra-Cytoplasmic Sperm Injection, *IVF* In vitro fertilization, *LH* Luteinizing hormone; *OCP* Oral contraceptive pills, *OHSS* Ovarian Hyper stimulation syndrome, *OS* Ovarian stimulation, *PCO* Polycystic ovaries, *PGD* Pre-implantation genetic diagnosis, *PGT* Pre-implantation genetic testing, *TVS* Transvaginal Sonography, *TESA* Testicular sperm aspiration, *U/S* Ultrasound, *β-hCG* Beta-human chorionic gonadotropin

### Outcome parameters

The main outcome measures were live birth rate per randomized patient, ongoing pregnancy (positive fetal heartbeat on ultrasound at 10-12 weeks of gestation) per randomized patient and clinical pregnancy rate (presence of gestational sac on ultrasound at a gestational age of 6-7 weeks) per randomized patient. Additional outcome measures were cumulative live birth rate (pregnancy achieved within 6 months after randomization), miscarriage rate, ectopic pregnancy rate, multiple pregnancy rate (presence of more than one gestational sac on transvaginal ultrasound), pain during the procedure using visual analogue scale (VAS) and adverse events (i.e. infection, dizziness, fever).

### Quality of included studies

The methodological characteristics of included studies were extracted and appraised while the risk of bias of individual RCTs was formally assessed using RoB-2 [49].

### Quantitative data synthesis

The dichotomous data results for each of the eligible for meta-analysis studies were expressed as risk ratio (RR) with 95% confidence intervals (CI) and they were analyzed according to the intention-to-treat principle. These results were combined for meta-analysis using the Mantel/Haenszel model when using the fixed effects model and the restricted maximum likelihood method with Hartung-Knapp-Sidik-Jonkman correction [50, 51] when using the random effects model (in case of high heterogeneity, i.e. I^2^≥50%). All results were combined for meta-analysis with the STATA Software (StataCorp. 2021. Stata Statistical Software: Release 17. College Station, TX: StataCorp LLC.). Statistical heterogeneity was estimated with the I^2^ statistic [52].

Prespecified subgroup analyses for live birth (being the most clinically important of the main outcomes) were performed according to a) the device used to perform endometrial scratching, b) the timing of the endometrial scratching, c) whether single or double endometrial scratching was performed, d) whether the population studied had previous failed IVF cycles or not, and d) the minimum number of previous failed IVF cycles of the population analyzed. This latter factor was also explored through meta-regression [53].

Statistical significance was set at a p level of 0.05. Publication bias was explored using the Harbord test [54]. A sensitivity analysis was performed for live birth, ongoing pregnancy and clinical pregnancy by excluding studies judged to be overall at high risk of bias according to RoB-2.

The certainty of evidence was assessed using the GRADEpro GDT (GRADEpro Guideline Development Tool [Software]. McMaster University and Evidence Prime, 2022. Available from gradepro.org) (Supplementary Table 1). For outcomes where a beneficial effect was suggested by the evidence, the number-needed-to treat (NNT) (i.e. number of patients required to receive the endometrial scratch in order for an additional person to either incur or avoid the event of interest) was also calculated to illustrate the impact and efficacy of endometrial injury.

## Results

**Fig. 1:**
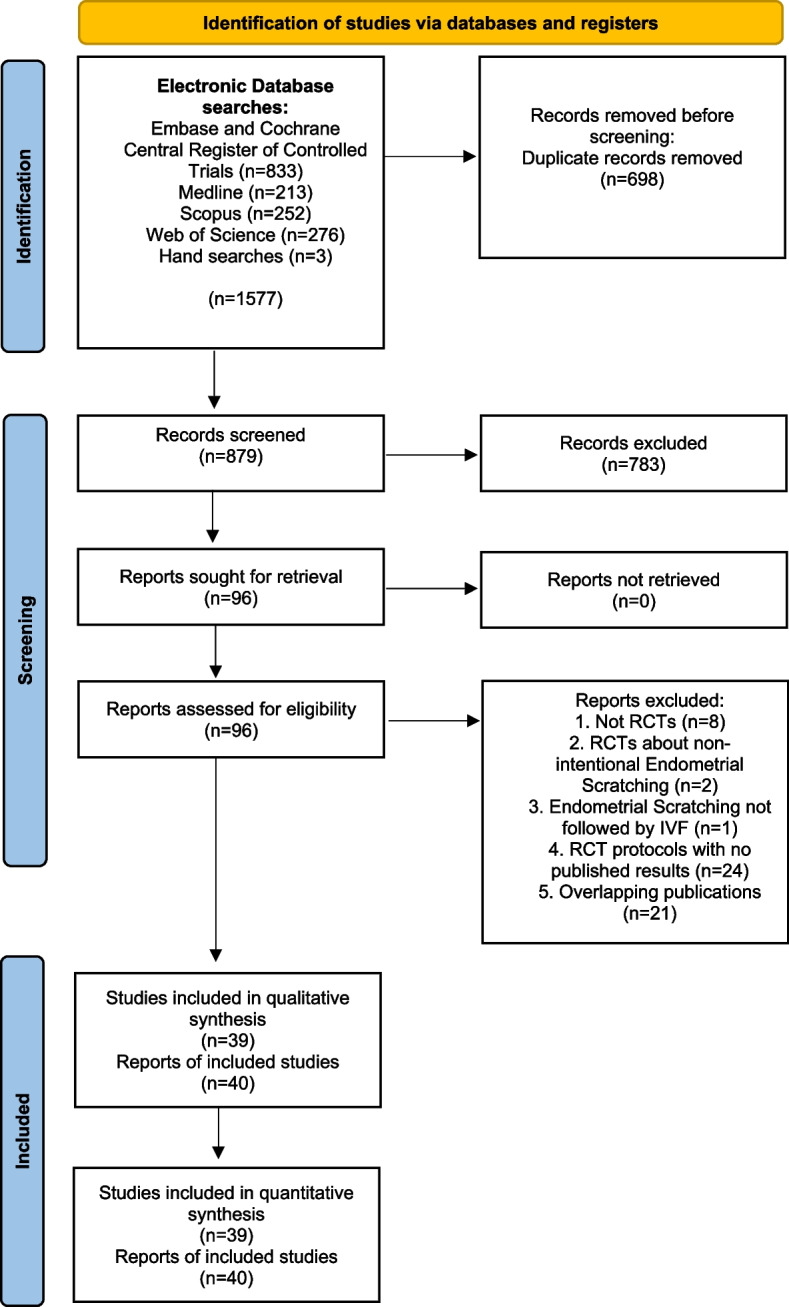
PRISMA Flow Chart

The literature search yielded 879 potentially relevant reports (Fig. 1). Subsequently, the titles of these manuscripts were examined, resulting in 222 potentially eligible publications. The abstracts of these studies were then examined and eventually 96 manuscripts that could provide data to answer the research question were identified. The full text of these studies was examined thoroughly, resulting in the inclusion of 40 publications, that represent 39 RCTs [5, 8, 11–45, 47, 48] (one report [46] contained post-hoc analyses of a previously published RCT [41] (Table 1). It should also be noted that Liu et al., [29] included four groups in their study (intervention and no intervention during the follicular and the luteal phase of the cycle preceding IVF) and therefore, we analyzed the follicular and the luteal phase arms of the study separately. Characteristics of the reports included in the systematic review appear in Tables 1 and 2. Eligible studies were published between 2008 and 2022. Randomization method was reported in 34 of the publications included, while allocation concealment method was reported in 19 of the studies included (Table 1). Most studies did not state clearly if the participants or those involved in the analysis were blinded to the type of intervention. Only 3 studies were reported to be single-blind and 3 were reported to be double-blind. Financial support was declared in 20 studies (Table 1). The largest study published so far on this issue was by Lensen et al. in 2019 [5]. The risk of bias assessment of the eligible studies is presented in Table 3. Overall, 9 studies [11–13, 15, 16, 23, 27, 28, 37] were deemed to be at high risk of bias (Supplementary Figures 1 & 2).

**Table 3:** Risk of Bias assessment of included studies (using RoB-2)

**Study**	**Domain 1**	**Domain 2**	**Domain 3**	**Domain 4**	**Domain 5**	**Overall**
Karim Zadeh 2008 [11]	Some concerns	High risk	High risk	Low risk	Some concerns	High risk
Karim Zadeh 2009 [12]	Some concerns	Some concerns	Low risk	Low risk	Some concerns	High risk
Karimzadeh 2010 [13]	Low risk	Low risk	Low risk	Low risk	Some concerns	High risk
Narvekar 2010 [14]	Low risk	Low risk	Low risk	Low risk	Some concerns	Some concerns
Safdarian 2011 [15]	Some concerns	Some concerns	Some concerns	Low risk	Some concerns	High risk
Baum 2012 [16]	Some concerns	Some concerns	Low risk	Low risk	Some concerns	High risk
Inal 2012 [17]	Some concerns	Low risk	Low risk	Low risk	Some concerns	Some concerns
Shohayeb 2012 [18]	Low risk	Some concerns	Low risk	Low risk	Some concerns	Some concerns
Nastri 2013 [19]	Low risk	Low risk	Low risk	Low risk	Low risk	Some concerns
Guven 2014 [20]	Low risk	Some concerns	Low risk	Low risk	Low risk	Some concerns
Yeung 2014 [21]	Low risk	Low risk	Low risk	Low risk	Low risk	Low risk
Gibreel 2015 [22]	High risk	Low risk	Low risk	Low risk	Low risk	Some concerns
Singh 2015 [23]	Some concerns	Low risk	Low risk	Low risk	Some concerns	High risk
Xu 2015 [24]	Some concerns	Low risk	Low risk	Low risk	Some concerns	Some concerns
Zhang 2015 [25]	Some concerns	Low risk	Low risk	Low risk	Some concerns	Some concerns
Aflatoonian 2016 [26]	Some concerns	Some concerns	Low risk	Low risk	Some concerns	Some concerns
Shahrokh-Tehraninejad 2016 [27]	Some concerns	High risk	Some concerns	Low risk	Some concerns	High risk
Zygula 2016 [28]	Some concerns	Some concerns	High risk	Low risk	Some concerns	High risk
Liu 2017 [29]	Some concerns	Low risk	Low risk	Low risk	Some concerns	Some concerns
Mak 2017 [30]	Low risk	Low risk	Low risk	Low risk	Low risk	Low risk
Tk 2017 [31]	Low risk	Low risk	Low risk	Low risk	Some concerns	Some concerns
Maged 2018 [32]	Low risk	Low risk	Low risk	Low risk	Low risk	Low risk
Pecorino 2018 [33]	Some concerns	Low risk	Low risk	Some concerns	Some concerns	Some concerns
Sherif 2018 [34]	Some concerns	Low risk	Low risk	Some concerns	Low risk	Some concerns
Eskew 2019 [35]	Low risk	Low risk	Low risk	Low risk	Some concerns	Some concerns
Frantz 2019 [36]	Low risk	Some concerns	Low risk	Low risk	Low risk	Some concerns
Gurgan 2019 [37]	Some concerns	High risk	High risk	Low risk	Some concerns	High risk
Hilton 2019 [38]	Low risk	Low risk	Low risk	Low risk	Low risk	Low risk
Lensen 2019 [5, 6]	Low risk	Low risk	Low risk	Low risk	Low risk	Low risk
Olesen 2019 [39]	Low risk	Low risk	Low risk	Some concerns	Low risk	Some concerns
Berntsen 2020 [40]	Some concerns	High risk	Some concerns	Low risk	Low risk	Some concerns
Izquierdo 2020 [41]	Some concerns	Low risk	Low risk	Low risk	Low risk	Low risk
Mackens 2020 [42]	Low risk	Some concerns	Low risk	Low risk	Low risk	Some concerns
Tang 2020 [43]	Low risk	Some concerns	Low risk	Low risk	Some concerns	Some concerns
Van Hoogenhuijze 2020 [8, 9]	Low risk	Low risk	Low risk	Low risk	Low risk	Low risk
Metwally 2021 [44]	Low risk	Low risk	Low risk	Low risk	Low risk	Low risk
Zahiri 2021 [45]	Some concerns	Some concerns	Low risk	Some concerns	Low risk	Some concerns
Noori 2022 [47]	Low risk	Low risk	Low risk	Some concerns	Some concerns	Some concerns
Turktekin 2022 [48]	Some concerns	Some concerns	Low risk	Low risk	Some concerns	Some concerns

### Meta-analysis

#### Live birth

**Fig. 2:**
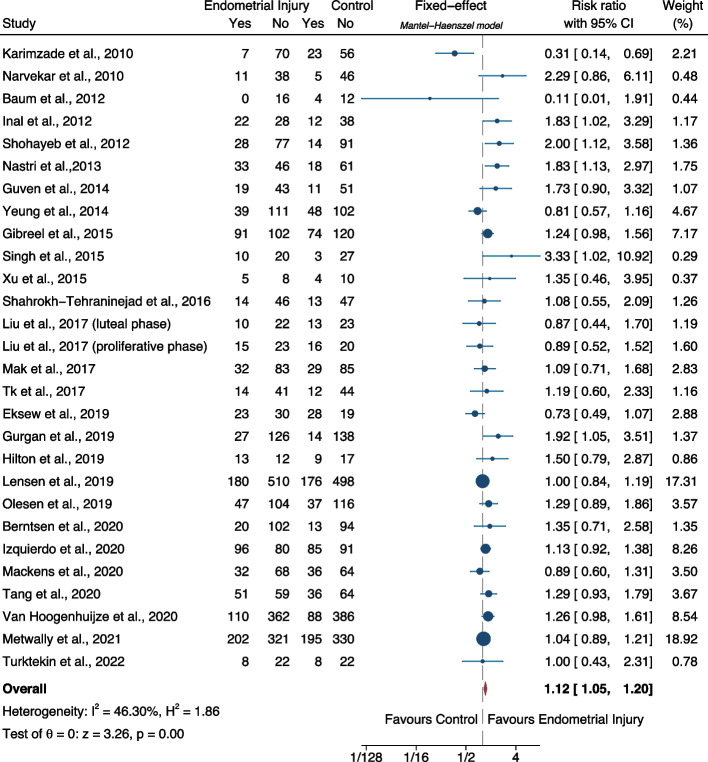
Forest plot presenting the risk ratio of live birth between women who had endometrial scratching prior to their embryo transfer and those who had a placebo/sham procedure or no intervention

A significantly higher probability of live birth was present in embryo transfer cycles after endometrial scratching as compared to placebo/sham or no intervention (risk ratio-RR: 1.12, 95% CI: 1.05– 1.20; fixed effects model; heterogeneity: I^2^=46.30%, 28 studies, 29 datasets, 7425 patients; low certainty; NNT: 30) (Fig. 2). Publication bias did not seem to be present (p=0.727). A sensitivity analysis excluding studies at high risk of bias [13, 15, 16, 23, 27, 37] did not materially change the results obtained (RR: 1.13, 95% CI: 1.05-1.21; fixed effects model; heterogeneity: I^2^=29.87%, 22 studies, 23 datasets; moderate certainty; NNT: 28) (Supplementary Figure 3).

#### Ongoing pregnancy

**Fig. 3:**
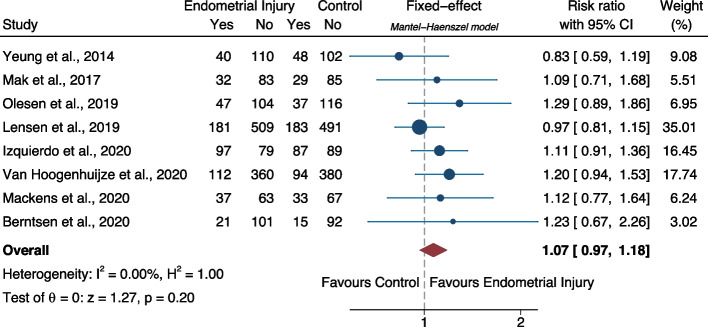
Forest plot presenting the risk ratio of ongoing pregnancy between women who had endometrial scratching prior to their embryo transfer and those who had a placebo/sham procedure or no intervention

A higher, but not significantly so, probability of ongoing pregnancy was present in embryo transfer cycles after endometrial scratching as compared to placebo/sham or no intervention (RR: 1.07, 95% CI: 0.98– 1.18; fixed effects model; heterogeneity: I^2^=27.44%, 11 studies, 11 datasets, 4515 patient; low certainty) (Fig. 3). Publication bias did not seem to be present (*p*=0.494). A sensitivity analysis excluding studies at high risk of bias did not materially change the results obtained (RR: 1.07, 95% CI: 0.97-1.18; fixed effects model; heterogeneity: I^2^=0.00%, 8 studies, 8 datasets; moderate certainty) (Supplementary Figure 4).

#### Clinical pregnancy

**Fig. 4:**
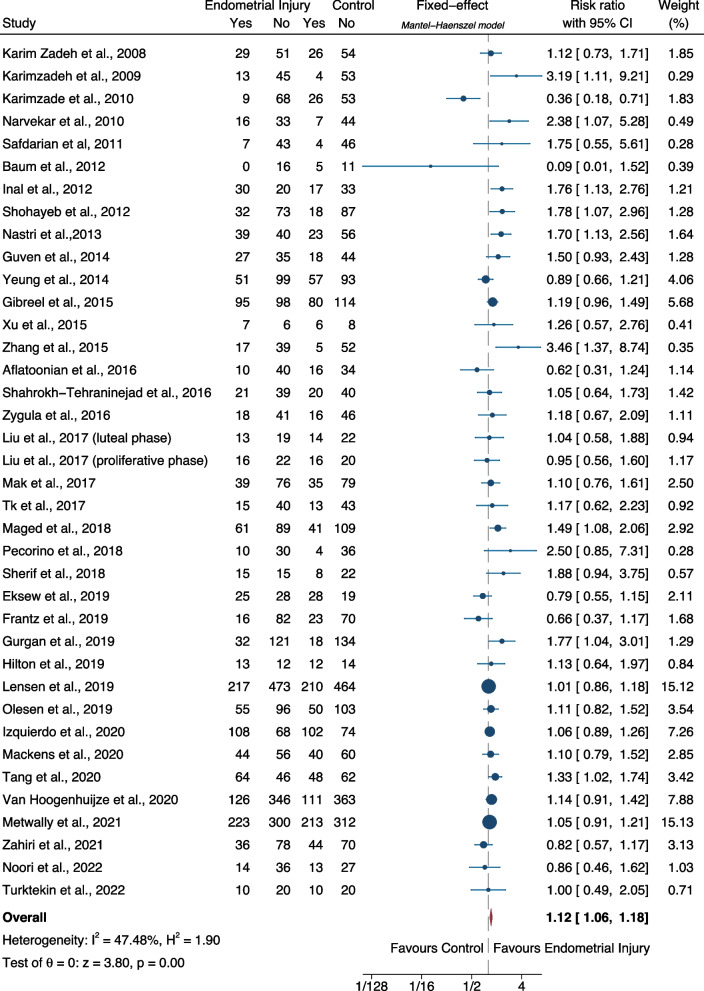
Forest plot presenting the risk ratio of clinical pregnancy between women who had endometrial scratching prior to their embryo transfer and those who had a placebo/sham procedure or no intervention

A significantly higher probability of clinical pregnancy was present in embryo transfer cycles after endometrial scratching as compared to placebo/sham or no intervention (RR: 1.12, 95% CI: 1.06– 1.18; fixed effects model; heterogeneity: I^2^=47.48%, 37 studies, 38 datasets, 8804 patients; low certainty; NNT: 27) (Fig. 4). Publication bias did not seem to be present (*p*=0.514). A sensitivity analysis excluding studies at high risk of bias did not materially change the results obtained (RR: 1.12, 95% CI: 1.05-1.19; fixed effects model; heterogeneity: I^2^=21.88%, 21 studies, 22 datasets; moderate certainty; NNT: 25) (Supplementary Figure 5).

#### Cumulative live birth

A higher, but not significantly so, probability of cumulative live birth was present in embryo transfer cycles after endometrial scratching as compared to placebo/sham or no intervention (RR: 1.11, 95% CI: 0.99–1.24; fixed effects model; heterogeneity: I^2^=0%, 2 studies, 1298 patients; very low certainty) (Supplementary Figure 6). Publication bias could not be assessed due to the small number of available studies.

#### Miscarriage

No significant difference in the probability of miscarriage was present in embryo transfer cycles after endometrial scratching as compared to placebo/sham or no intervention (RR: 0.89, 95% CI: 0.75–1.06; fixed effects model; heterogeneity: I^2^=0%, 24 studies, 25 datasets, 2568 patients; low certainty) (Supplementary Figure 7). Publication bias did not seem to be present (*p*=0.432).

#### Ectopic pregnancy

No significant difference in the probability of ectopic pregnancy was present in embryo transfer cycles after endometrial scratching as compared to placebo/sham or no intervention (RR: 1.02, 95% CI: 0.46– 2.27; fixed effects model; heterogeneity: I^2^=0%, 8 studies, 9 datasets, 1219 patients; very low certainty) (Supplementary Figure 8). Publication bias did not seem to be present (*p*=0.148).

#### Multiple pregnancy

No significant difference in the probability of multiple pregnancy was present in embryo transfer cycles after endometrial scratching as compared to placebo/sham or no intervention (RR: 1.11, 95% CI: 0.92–1.35; fixed effects model; heterogeneity: I^2^=25.68%, 17 studies, 18 datasets, 1974 patients; low certainty) (Supplementary Figure 9). Publication bias did not seem to be present (*p*=0.482).

#### Adverse events

##### Pain

Five studies [4, 8, 19, 36, 44] reported pain in the endometrial scratching group with VAS scores ranging from 3.5 to 6.4. Only one study (158 patients) provided VAS scores both in the endometrial scratching group and the control group (sham procedure) indicating higher pain scores (6.42, SD (2.35) vs 1.82, SD (1.52); *P* < 0.001) in women who had the endometrial scratching [19].

##### Bleeding

In patients allocated to endometrial scratching, bleeding was reported in a proportion of them in four studies [5, 8, 33, 42], while in further 8 studies [13, 19, 23, 29, 38, 39, 41, 43] no patients experienced bleeding after endometrial scratching The remaining studies did not report on this adverse event.

##### Infection

In patients allocated to endometrial scratching no infections were observed in 11 studies [5, 8, 13, 19, 23, 29, 38, 39, 41–43], while the remaining studies did not report on this adverse event.

##### Dizziness

In patients allocated to endometrial scratching, dizziness was not observed in 10 studies [8, 13, 19, 23, 29, 38, 39, 41–43] while in a single study [5] 7 out of 690 patients (~1%) who underwent endometrial scratching experienced this adverse event.

##### Fever

In patients allocated to endometrial scratching, fever was not observed in 10 studies [5, 13, 19, 23, 29, 39, 41–43] while in a single study [8] 3 out of 742 patients (0.6%) who underwent endometrial scratching experienced this adverse event.

### Subgroup analyses

#### Type of instrument used to perform the endometrial injury

Pipelle-type catheters were used for endometrial scratching in 29 trials, while Novak curette was the tool of choice in 3 trials. A variety of other instruments were used for endometrial injury in the remaining studies (Table 2). The type of instrument used to perform endometrial scratching did not appear to be associated with the effect size observed (test for subgroup differences: *p*=0.13).

#### Timing of the endometrial injury

Endometrial scratching was performed during the cycle preceding IVF treatment in 33 RCTs (Table 2). In a single study, endometrial scratching was performed from day 3 of the cycle preceding embryo transfer until day 3 of the treatment cycle [5]. In 3 of the eligible RCTs, endometrial scratching was performed during the follicular phase of the cycle, while in further 3 RCTs it was performed on the day of oocyte retrieval (Table 2).

A subgroup analysis based on the time endometrial scratching was performed (in the preceding cycle, in the actual embryo transfer cycle or in either of the two) suggested significant difference between the subgroups (*p*=0.04) (Supplementary Figure 10). Studies in which the endometrial scratching was performed during the preceding cycle showed a pooled RR: 1.18 (95% CI:1.09-1.27; moderate certainty; NNT: 21), whereas studies in which the endometrial scratching was performed during the embryo transfer cycle showed a pooled RR: 0.87 (95% CI: 0.67-1.15; low certainty).

#### Single of double endometrial injury

Single or double endometrial scratching was performed in 34 and 5 of the eligible RCTs, respectively (Table 2). A subgroup analysis between studies with single and those with double endometrial scratching did not suggest a significant difference in the probability of live birth (*p*=0.27).

#### History of previous failed IVF cycles

A subgroup analysis according to whether the population evaluated in each study had experienced previous IVF failures or not suggested a significant difference between subgroups (*p*<0.001). The highest effect size was observed in studies which randomized patients with previous IVF failures (RR: 1.35, 95% CI: 1.20-1.53, fixed effects model, heterogeneity: I^2^=0.06%, 13 studies, 13 datasets, 2627 patients; moderate certainty; NNT: 14) (Supplementary Figure 11).

A further subgroup analysis according to the minimum number of previous IVF failures (0,1,2 and 3) also confirmed a significant difference between subgroups (*p*=0.04), with the largest effect size observed in studies that included patients with at least 3 failed IVF cycles (RR: 1.70, 95% CI: 1.14-2.54, fixed effects model; heterogeneity: I^2^=49.75%, 3 studies, 547 patients; low certainty; NNT: 12) (Supplementary Figure 12). Finally, a meta-regression performed using the minimum number of previous failed as an independent variable, suggested a positive significant association with the risk ratio of live birth in the included studies (coeff: 0.18, 9% CI: 0.06-0.31; *p*=0.004).

## Discussion

The aim of this review was to evaluate the impact of endometrial scratching on reproductive outcomes in women undergoing IVF compared to no intervention or sham intervention and to clarify if certain subgroups of patients could benefit more from it. Following the pooled analysis of 39 RCTs including ~9000 patients, this updated systematic review and meta-analysis suggests that endometrial scratching, compared to no or a sham intervention, can improve live birth and clinical pregnancy rates after IVF by a relative increase of 12%. This finding persisted in the sensitivity analysis performed where studies deemed to be at high risk of bias were excluded. On the other hand, this systematic review could not detect a significant positive effect on ongoing pregnancy rates, however, that analysis included only 11 RCTs and therefore a type II error cannot be excluded.

The most recent Cochrane systematic review and meta-analysis has reviewed 37 studies published by June 2020 and eventually pooled data only from eight studies deemed to be at low risk of bias including in total 4402 patients. Regarding live birth, their pooled analysis did not detect a significant effect of endometrial scratching on live birth rates (odds ratio: 1.12, 95% CI: 0.98-1.28). Nevertheless, given the effect size observed, which suggests a potential (non-significant) benefit, the authors concluded that it is unclear whether a benefit truly exists. It should be noted that the lack of statistical significance could represent a type II error given the limited number of studies analyzed, which was a post-hoc decision and a departure from the review protocol. This post-hoc decision creates methodological challenges when interpreting the results of the Cochrane review, particularly since the Cochrane Handbook for Systematic Reviews of Interventions suggests that sensitivity analyses are used to check the robustness of results by excluding studies at high risk of bias [55]. The present systematic review and meta-analysis has reviewed and analyzed the entire body of available evidence published until 2023 following established guidelines on dealing with potential bias.

Furthermore, the present systematic review has analyzed several potential effect moderators via subgroup analyses and meta-regression. These analyses suggested that the pooled effect size of studies where the endometrial injury was performed in the cycle before the embryo transfer was higher than that observed in studies where endometrial injury was performed for some or all patients during the actual embryo transfer cycle. The most recent Cochrane review, due to the restriction of the analysis to 8 RCTs, was not able to perform such a comparison. The implications of this finding can be significant as it has been argued [8, 10] that the timing of the biopsy is a clinically important variable.

Another important finding of the subgroup analyses is the potential significance of the type of population included in the eligible RCTs. The subgroup analysis comparing studies where patients recruited had previous failed IVF cycles or not (or there was a mix of both), strongly suggested that the intervention is far more likely to have a beneficial effect on patients with previous failed IVF cycles. This finding was confirmed in further subgroup analyses based on the minimum number of previous failed IVF cycles and the relevant meta-regression, both of which suggested that the higher the number of previous failed IVF cycles, the higher the risk ratio observed, implying a stronger benefit of endometrial scratching. The explanation of this finding could lie in the progressively better selection of poorer prognosis patients, more likely to have an endometrial issue who can benefit from the intervention, as it was suggested in the original report by Barash et al [1]. Other authors have also supported that hysteroscopy combined with endometrial injury is beneficial for patients with repeated IVF failures [56, 57]. The beneficial effect of endometrial injury in patients with prior failed embryo transfers has also previously been reported in a meta-analysis published in 2018 [3]. The latest Cochrane systematic review did not identify an association with previous IVF failures, however, the limited number of studies analyzed could once again have limited the statistical power of this test.

The subgroup analysis depending on whether endometrial scratching was performed once or twice on the same patient did not show any difference between the two subgroups compared. Moreover, the subgroup analysis depending on the type of device used to perform endometrial scratching did not suggest that this is important for the probability of live birth. The most recent Cochrane review did not address the same clinical questions, although it did compare higher with lower intensity of endometrial injury and failed to detect a difference in the effect sizes between the two methods. These findings suggest that performing endometrial scratching once with a pipelle catheter is likely to be sufficient for a beneficial effect to be elicited.

In terms of the remaining secondary outcomes, the present systematic review and meta-analysis did not find a difference in ectopic pregnancy, miscarriage and multiple pregnancy rates between women who had embryo transfer after endometrial scratching and those who had not. This is in agreement with what has been previously reported [7]. Other important outcomes in the evaluation of endometrial scratching are adverse events such as pain, bleeding, dizziness, infection and fever. A comparative assessment of the incidence of such adverse events would only be possible in studies that performed a sham procedure in the control group. In the present systematic review only one study [19] provided such data indicating higher pain experienced in women who had endometrial scratching compared to those who had the sham procedure. However, what might be of more clinical relevance is the incidence of such adverse events in women undergoing endometrial scratching. The incidence of pain and/or bleeding varied widely in the included studies from 0% to 75%, likely reflecting differences in the methodologies used to capture these adverse events. Reassuringly, infection, dizziness and fever after endometrial biopsy was reported to be rare, with only one out of the eleven studies reporting dizziness [5] or fever [8] at a rate of ~1%, while the remaining 10 studies reported that none of the patients experienced these adverse events.

An individual participant data meta-analysis (IPD-MA) on the potential benefit of endometrial injury was recently published confirming that live birth rates are higher after endometrial injury compared to no scratch/sham procedure (odds ratio: 1.29, 95% CI: 1.02-1.64). Despite the obvious methodological advantages of an IPD-MA, the researchers were only able to include 13 RCTs (*n*=4112 participants) which is <50% of the sample size included in the present meta-analysis. This might explain why a significant interaction effect with the number of previous failed embryo transfers was not detected in the IPD-MA, something the present meta-analysis has been able to show by analyzing the total body of published evidence.

It should be noted that the present systematic review is also characterized by limitations such as the clinical heterogeneity in the eligible studies regarding the population studied and the method used to implement endometrial scratching that should be taken into consideration when interpreting the results obtained. To facilitate this interpretation several subgroup analyses have been performed to identify the potential moderating effect of these factors. The quality of the eligible studies also varied with some studies being graded as at high risk of bias. A sensitivity analysis was performed by excluding these studies and the results obtained were not materially different to the main analysis. Finally, most of the included studies did not seem to capture the adverse effects of endometrial scratching, and this information is important when counselling patients about the potential benefits and risks of the intervention.

The present systematic review and meta-analysis represents an updated critical appraisal of an intervention that has been extensively used in clinical practice during the last decade. Its results are able to inform clinicians and patients regarding important questions including, which patients might benefit from endometrial scratching, what is the optimal method of endometrial scratching and when it should be performed. Nevertheless, it is also evident from the present work that further data is required to confirm or rebut its findings and based on this systematic review future clinical research should focus on endometrial scratching during the cycle prior to IVF in patients with multiple previous IVF failures. Concurrently, future basic research needs to identify a plausible mechanism through which endometrial scratching exerts its observed beneficial effect.

In conclusion, the present systematic review and meta-analysis suggests that endometrial scratching during the menstrual cycle prior to IVF can lead to a higher probability of live birth in patients with previous IVF failures and that this effect seems to be greater in patients with more IVF failures.

### Supplementary Information

**Additional file 1:** **Supplementary Figure 1.** Summary plot of the risk of bias assessment.

**Additional file 2:** **Supplementary Figure 2.** Traffic lights plot of the risk of bias assessment.

**Additional file 3:** **Supplementary Figure 3.** Forest plot presenting the sensitivity analysis (by excluding studies at high risk of bias) on the risk ratio of live birth raes between women who had endometrial scratching prior to their embryo transfer and those who had a placebo/sham procedure or no intervention.

**Additional file 4:** **Supplementary Figure 4.** Forest plot presenting the sensitivity analysis (by excluding studies at high risk of bias) on the risk ratio of ongoing pregnancy between women who had endometrial scratching prior to their embryo transfer and those who had a placebo/sham procedure or no intervention.

**Additional file 5:** **Supplementary Figure 5.** Forest plot presenting the sensitivity analysis (by excluding studies at high risk of bias) on the risk ratio of clinical pregnancy between women who had endometrial scratching prior to their embryo transfer and those who had a placebo/sham procedure or no intervention.

**Additional file 6:** **Supplementary Figure 6.** Forest plot presenting the risk ratio of cumulative live birth between women who had endometrial scratching prior to their embryo transfer and those who had a placebo/sham procedure or no intervention.

**Additional file 7:** **Supplementary Figure 7.** Forest plot presenting the risk ratio of miscarriage between women who had endometrial scratching prior to their embryo transfer and those who had a placebo/sham procedure or no intervention.

**Additional file 8:** **Supplementary Figure 8.** Forest plot presenting the risk ratio of ectopic pregnancy between women who had endometrial scratching prior to their embryo transfer and those who had a placebo/sham procedure or no intervention.

**Additional file 9:** **Supplementary Figure 9.** Forest plot presenting the risk ratio of multiple pregnancy between women who had endometrial scratching prior to their embryo transfer and those who had a placebo/sham procedure or no intervention.

**Additional file 10:** **Supplementary Figure 10.** Forest plot presenting the subgroup analysis of the risk ratio of live birth between women who had endometrial scratching prior to their embryo transfer and those who had a placebo/sham procedure or no intervention according to the timing of endometrial injury.

**Additional file 11:** **Supplementary Figure 11.** Forest plot presenting the subgroup analysis of the risk ratio of live birth between women who had endometrial scratching prior to their embryo transfer and those who had a placebo/sham procedure or no intervention according to the whether the population included had a history of previous IVF failures or not.

**Additional file 12:** **Supplementary Figure 12.** Forest plot presenting the subgroup analysis of the risk ratio of live birth between women who had endometrial scratching prior to their embryo transfer and those who had a placebo/sham procedure or no intervention according to the minimum number of previous IVF failures.

**Additional file 13:** **Supplementary Table 1.** Certainty assessment of the available evidence using the GRADEPro Guideline Development Tool.

## Abbreviations

ART: Assisted reproductive technologies
CI: Confidence interval
IVF: In vitro fertilization
RCT: Randomized controlled trial
RR: Risk ratio
VAS: Visual analogue scale
