# Supplementary figures and images for: The role of endometrial scratching prior to in vitro fertilization: an updated systematic review and meta-analysis
Source: Reprod Biol Endocrinol. 2023 Oct 2;21:89. doi: 10.1186/s12958-023-01141-2 (PMC10544419; doi:10.1186/s12958-023-01141-2)

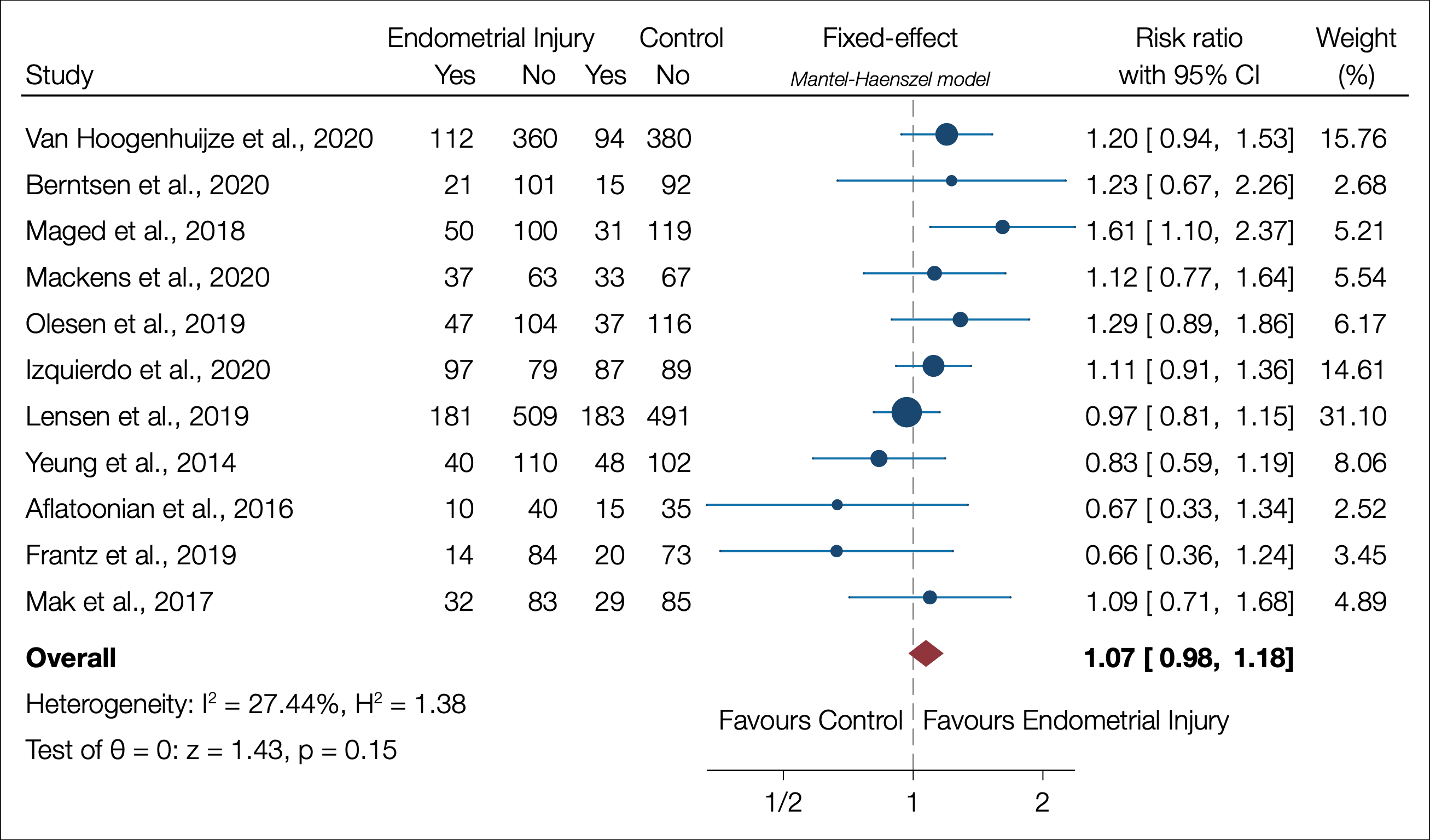

Supplement: Supplementary file 4 — Additional file 4: Supplementary Figure 4. Forest plot presenting the sensitivity analysis (by excluding studies at high risk of bias) on the risk ratio of ongoing pregnancy between women who had endometrial scratching prior to their embryo transfer and those who had a placebo/sham procedure or no intervention. [file 12958_2023_1141_MOESM4_ESM.tif]
