# Supplementary material for: The role of endometrial scratching prior to in vitro fertilization: an updated systematic review and meta-analysis
Source: Reprod Biol Endocrinol. 2023 Oct 2;21:89. doi: 10.1186/s12958-023-01141-2 (PMC10544419; doi:10.1186/s12958-023-01141-2)
Supplement: Supplementary file 13 — Additional file 13: Supplementary Table 1. Certainty assessment of the available evidence using the GRADEPro Guideline Development Tool. [file 12958_2023_1141_MOESM13_ESM.docx]

**Supplementary Table 1. Certainty assessment of the available evidence using the GRADEPro Guideline Development Tool**

| **Certainty assessment** | | | | | | | **№ of patients** | | **Effect** | | **Certainty** | **Importance** |
| --- | --- | --- | --- | --- | --- | --- | --- | --- | --- | --- | --- | --- |
| **№ of studies** | **Study design** | **Risk of bias** | **Inconsistency** | **Indirectness** | **Imprecision** | **Other considerations** | **Endometrial scratching prior to an IVF cycle** | **No intervention or placebo/sham intervention** | **Relative (95% CI)** | **Absolute (95% CI)** |  |  |
| **Live Birth** | | | | | | | | | | | | |
| 28 | randomised trials | serious | serious^a^ | not serious | not serious | none | 1159/3729 (31.1%) | 1024/3696 (27.7%) | **RR 1.12** (1.05 to 1.20) | **33 more per 1,000** (from 14 more to 55 more) | ⨁⨁◯◯ Low | CRITICAL |
| **Live birth- Sensitivity analysis** | | | | | | | | | | | | |
| 22 | randomised trials | not serious | serious^a^ | not serious | not serious | none | 1101/3393 (32.4%) | 967/3359 (28.8%) | **RR 1.13** (1.05 to 1.21) | **37 more per 1,000** (from 14 more to 60 more) | ⨁⨁⨁◯ Moderate | CRITICAL |
| **Ongoing pregnancy** | | | | | | | | | | | | |
| 11 | randomised trials | serious | serious^a^ | not serious | not serious | none | 641/2274 (28.2%) | 592/2241 (26.4%) | **RR 1.07** (0.98 to 1.18) | **18 more per 1,000** (from 5 fewer to 48 more) | ⨁⨁◯◯ Low | CRITICAL |
| **Ongoing pregnancy- Sensitivity analysis** | | | | | | | | | | | | |
| 8 | randomised trials | not serious | serious^a^ | not serious | not serious | none | 567/1976 (28.7%) | 526/1948 (27.0%) | **RR 1.07** (0.97 to 1.18) | **19 more per 1,000** (from 8 fewer to 49 more) | ⨁⨁⨁◯ Moderate | CRITICAL |
| **Clinical Pregnancy** | | | | | | | | | | | | |
| 37 | randomised trials | serious | serious^a^ | not serious | not serious | none | 1573/4412 (35.7%) | 1401/4392 (31.9%) | **RR 1.12** (1.06 to 1.18) | **38 more per 1,000** (from 19 more to 57 more) | ⨁⨁◯◯ Low | CRITICAL |
| **Clinical Pregnancy- Sensitivity analysis** | | | | | | | | | | | | |
| 21 | randomised trials | not serious | serious^a^ | not serious | not serious | none | 1265/3271 (38.7%) | 1128/3262 (34.6%) | **RR 1.12** (1.05 to 1.19) | **41 more per 1,000** (from 17 more to 66 more) | ⨁⨁⨁◯ Moderate | CRITICAL |
| **Cumulative live birth (follow-up: 12 months)** | | | | | | | | | | | | |
| 2 | randomised trials | serious^b^ | serious^b^ | not serious | serious^b^ | none | 325/648 (50.2%) | 294/650 (45.2%) | **RR 1.11** (0.99 to 1.24) | **50 more per 1,000** (from 5 fewer to 109 more) | ⨁◯◯◯ Very low | IMPORTANT |
| **Miscarriage** | | | | | | | | | | | | |
| 24 | randomised trials | serious | serious^a^ | not serious | not serious | none | 204/1396 (14.6%) | 209/1268 (16.5%) | **RR 0.89** (0.75 to 1.06) | **18 fewer per 1,000** (from 41 fewer to 10 more) | ⨁⨁◯◯ Low | IMPORTANT |
| **Ectopic pregnancy** | | | | | | | | | | | | |
| 8 | randomised trials | serious | serious^a^ | not serious | serious^c^ | none | 10/629 (1.6%) | 9/590 (1.5%) | **RR 1.02** (0.46 to 2.27) | **0 fewer per 1,000** (from 8 fewer to 19 more) | ⨁◯◯◯ Very low | IMPORTANT |
| **Multiple pregnancy** | | | | | | | | | | | | |
| 17 | randomised trials | serious | serious^d^ | not serious | not serious | none | 171/1069 (16.0%) | 138/1001 (13.8%) | **RR 1.11** (0.92 to 1.35) | **15 more per 1,000** (from 11 fewer to 48 more) | ⨁⨁◯◯ Low | IMPORTANT |
| **Endometrial scratching during the cycle preceding IVF (subgroup analysis)** | | | | | | | | | | | | |
| 21 | randomised trials | serious | not serious | not serious | not serious | none | 908/2757 (32.9%) | 766/2737 (28.0%) | **RR 1.18** (1.09 to 1.27) | **50 more per 1,000** (from 25 more to 76 more) | ⨁⨁⨁◯ Moderate | CRITICAL |
| **Endometrial scratching during the embryo transfer cycle (subgroup analysis)** | | | | | | | | | | | | |
| 5 | randomised trials | serious | serious^d^ | not serious | not serious | none | 71/282 (25.2%) | 82/285 (28.8%) | **RR 0.87** (0.67 to 1.15) | **37 fewer per 1,000** (from 95 fewer to 43 more) | ⨁⨁◯◯ Low | CRITICAL |
| **History of previous IVF failures (subgroup analysis)** | | | | | | | | | | | | |
| 13 | randomised trials | serious | not serious | not serious | not serious | none | 445/1566 (28.4%) | 325/1548 (21.0%) | **RR 1.35** (1.20 to 1.53) | **73 more per 1,000** (from 42 more to 111 more) | ⨁⨁⨁◯ Moderate | CRITICAL |
| **No history of previous IVF failures (subgroup analysis)** | | | | | | | | | | | | |
| 3 | randomised trials | serious | not serious | not serious | serious^b^ | none | 246/655 (37.6%) | 235/659 (35.7%) | **RR 1.05** (0.91 to 1.21) | **18 more per 1,000** (from 32 fewer to 75 more) | ⨁⨁◯◯ Low | CRITICAL |
| **History of at least 3 previous failed IVF cycles (subgroup analysis)** | | | | | | | | | | | | |
| 3 | randomised trials | serious | not serious | not serious | serious^b^ | none | 55/274 (20.1%) | 32/273 (11.7%) | **RR 1.70** (1.14 to 2.54) | **82 more per 1,000** (from 16 more to 181 more) | ⨁⨁◯◯ Low | CRITICAL |
| **History of at least 2 previous failed IVF cycles (subgroup analysis)** | | | | | | | | | | | | |
| 1 | randomised trials | very serious | not serious | not serious | very serious^b^ | none | 14/60 (23.3%) | 13/60 (21.7%) | **RR 1.08** (0.55 to 2.09) | **17 more per 1,000** (from 97 fewer to 236 more) | ⨁◯◯◯ Very low | CRITICAL |
| **History of at least 1 previous failed IVF cycle (subgroup analysis)** | | | | | | | | | | | | |
| 8 | randomised trials | serious | not serious | not serious | not serious | none | 325/1122 (29.0%) | 244/1115 (21.9%) | **RR 1.30** (1.16 to 1.53) | **66 more per 1,000** (from 35 more to 116 more) | ⨁⨁⨁◯ Moderate | CRITICAL |
| **No history of previous failed IVF cycles (subgroup analysis)** | | | | | | | | | | | | |
| 3 | randomised trials | serious | not serious | not serious | serious^b^ | none | 316/725 (43.6%) | 235/639 (36.8%) | **RR 1.05** (0.91 to 1.21) | **18 more per 1,000** (from 33 fewer to 77 more) | ⨁⨁◯◯ Low | CRITICAL |

**CI:** confidence interval; **RR:** risk ratio

#### Explanations

a. Differences in the clinical protocol of endometrial injury and the population included in the individual trials

b. Small number of available studies

c. Wide 95% Confidence Intervals

d. Inconsistency across the results of pooled studies
